# Reduction of product composition variability using pooled microbiome ecosystem therapy and consequence in two infectious murine models

**DOI:** 10.1128/aem.00016-24

**Published:** 2024-04-23

**Authors:** Julie Reygner, Johanne Delannoy, Marie-Thérèse Barba-Goudiaby, Cyrielle Gasc, Benoît Levast, Enora Gaschet, Laurent Ferraris, Stéphane Paul, Nathalie Kapel, Anne-Judith Waligora-Dupriet, Frederic Barbut, Muriel Thomas, Carole Schwintner, Bastien Laperrousaz, Nathalie Corvaïa

**Affiliations:** 1 MaaT Pharma, Lyon, France; 2 UMR-S 1139, INSERM, Université Paris Cite, Paris, France; 3 UMR1319, Micalis Institute, INRAE, AgroParisTech, Université Paris-Saclay, Jouy-en-Josas, France; 4 Team GIMAP, Centre International de Recherche en Infectiologie, Université Jean Monnet, Saint-Etienne, France; 5 Inserm, Université Claude Bernard Lyon, Lyon, France; 6 CIC 1408 Inserm Vaccinology, University Hospital of Saint-Etienne, Saint-Etienne, France; 7 Immunology Department, iBiothera Reference Center, University Hospital of Saint-Etienne, Saint-Etienne, France; 8 Service de Coprologie fonctionnelle, Hôpital de la Pitié-Salpêtrière-Charles Foix, AP-HP, Paris, France; 9 National Reference Laboratory for Clostridioides difficile, Saint-Antoine Hospital, Assistance Publique-Hôpitaux de Paris, Paris, France; 10 The European Society of Clinical Microbiology and Infectious Diseases Study Group for Clostridioides difficile, Basel, Switzerland; University of Illinois Urbana-Champaign, Urbana, Illinois, USA

**Keywords:** gut microbiota, dysbiosis, fecal microbiota transfer, pooling strategy, infectious diseases, microbiotherapy

## Abstract

Growing evidence demonstrates the key role of the gut microbiota in human health and disease. The recent success of microbiotherapy products to treat recurrent *Clostridioides difficile* infection has shed light on its potential in conditions associated with gut dysbiosis, such as acute graft-versus-host disease, intestinal bowel diseases, neurodegenerative diseases, or even cancer. However, the difficulty in defining a “good” donor as well as the intrinsic variability of donor-derived products’ taxonomic composition limits the translatability and reproducibility of these studies. Thus, the pooling of donors’ feces has been proposed to homogenize product composition and achieve higher taxonomic richness and diversity. In this study, we compared the metagenomic profile of pooled products to corresponding single donor-derived products. We demonstrated that pooled products are more homogeneous, diverse, and enriched in beneficial bacteria known to produce anti-inflammatory short chain fatty acids compared to single donor-derived products. We then evaluated pooled products’ efficacy compared to corresponding single donor-derived products in *Salmonella* and *C. difficile* infectious mouse models. We were able to demonstrate that pooled products decreased pathogenicity by inducing a structural change in the intestinal microbiota composition. Single donor-derived product efficacy was variable, with some products failing to control disease progression. We further performed *in vitro* growth inhibition assays of two extremely drug-resistant bacteria, *Enterococcus faecium* vanA and *Klebsiella pneumoniae* oxa48, supporting the use of pooled microbiotherapies. Altogether, these results demonstrate that the heterogeneity of donor-derived products is corrected by pooled fecal microbiotherapies in several infectious preclinical models.

**IMPORTANCE:**

Growing evidence demonstrates the key role of the gut microbiota in human health and disease. Recent Food and Drug Administration approval of fecal microbiotherapy products to treat recurrent *Clostridioides difficile* infection has shed light on their potential to treat pathological conditions associated with gut dysbiosis. In this study, we combined metagenomic analysis with *in vitro* and *in vivo* studies to compare the efficacy of pooled microbiotherapy products to corresponding single donor-derived products. We demonstrate that pooled products are more homogeneous, diverse, and enriched in beneficial bacteria compared to single donor-derived products. We further reveal that pooled products decreased *Salmonella* and *Clostridioides difficile* pathogenicity in mice, while single donor-derived product efficacy was variable, with some products failing to control disease progression. Altogether, these findings support the development of pooled microbiotherapies to overcome donor-dependent treatment efficacy.

## INTRODUCTION

Over the last decades, the development of metagenomic tools has accelerated research on microbial communities, drastically improving knowledge on intestinal microbiota composition and function. The gut microbiota has proven to be essential to its host for the fermentation of non-digestible substrates, the maintenance of gut barrier integrity, the regulation of immune system homeostasis, and for protection against pathogens (1). Meanwhile, alteration of the gut microbiota composition and function, called dysbiosis, was also found in a number of pathological conditions. These findings led researchers to develop therapies aiming at restoring gut microbiota integrity and function for the treatment or prophylaxis of many diseases. The best example is the recent success of fecal microbiota transfer (FMT) for the treatment of recurrent *Clostridioides difficile* infection (rCDI), achieving an outstanding remission rate of 85%–95%, though the exact mechanism of action remains unclear (2–4). These results led to the first Food and Drug Administration (FDA) approval of a microbiotherapy product as well as its recommendation by the European and North American medical societies (5). The enthusiasm for microbiotherapies led to a surge in clinical research in a growing number of indications, such as infectious diseases (6–8), gastrointestinal diseases (9, 10), metabolic disorders (11, 12), auto-immune and inflammatory diseases (13, 14), neurological disorders (15), liver diseases (16), kidney diseases (17), acute gastrointestinal graft-versus-host (aGvHD) disease (18), and cancers (19–22). Although FMT is becoming standardized as part of the European SoHO regulation (23), the protocols for donor screening (24), product manufacturing, and administration differ according to local procedures (25). In addition, the difficulty in defining a “good” donor as well as the intrinsic variability of donor-derived products’ taxonomic composition limits the translatability and reproducibility of these studies (26). Indeed, it has been demonstrated that differences in donor-derived stool composition translate to major changes in engraftment dynamics, directly affecting treatment efficacy in gastrointestinal disorders (27–29). In particular, comparing the gut microbiota profiles of different donors has revealed that microbial diversity is a reliable predictor for FMT success (30). Thus, the pooling of several donors’ feces has been proposed to homogenize product composition, achieve higher taxonomic richness, and enrich specific bacterial genera with health benefits such as butyrate-producing bacteria (31–33). The accumulation of evidence shows that the FMT procedure is safe (34) despite a few serious incidents such as the transfer of resistant bacteria leading to the death of one immunocompromised patient, which could have been avoided by rigorous donor screening (35). Recently, we demonstrated the safety of pooled allogeneic fecal microbiotherapy in a population of highly immunocompromised steroid refractory gastrointestinal-GvHD (SR-GI-GvHD) patients in a phase II trial (18). An improved response rate was also observed in a cohort of 81 patients treated with pooled products in the compassionate use/expanded access program. We further evidenced that microbiota engraftment was improved when increasing the number of donors who contributed to the received pooled product (18, 36). In line, we performed a systematic meta-analysis demonstrating the superiority of pooled fecal microbiotherapy compared to single donor-derived products to achieve a clinical benefit in ulcerative colitis patients (37). The aim of this study was to evaluate the anti-infectious effect of the two microbiotherapy formulations, i.e., the microbiome of human single donor-derived products and corresponding pooled products in two well-established preclinical mouse models of infection with *Salmonella enterica* and *Clostridioides difficile*. We further compared the growth inhibition activity of single donor-derived products and corresponding pooled products against *C. difficile* and two extremely drug-resistant bacteria, *Enterococcus faecium* vanA and *Klebsiella pneumoniae* oxa48, through an *in vitro* culture assay.

## MATERIALS AND METHODS

### Test substance

The test substances used in this study were feces from different single healthy donors and related pooled products manufactured by MaaT Pharma, Lyon, France. All products were screened to validate the absence of pathogens using Filmarray Gastro-Intestinal Panel. Single donor-derived products were formulated as homogenized single-donation fecal microbiota suspensions. As previously described by Burz and colleagues (38), each individual donation was processed by adding 4 mL of MaaT Pharma’s proprietary diluent per gram of stool. Each fecal pooled product was formulated as a homogenized pooled fecal microbiota suspension from five to nine different donors. All products were stored as frozen suspensions at −80°C until use.

### Infectious murine models

All animal experiments for *Salmonella* murine model were performed at PLEXAN (Platform for Experiments and Analysis, Faculty of Medicine, Université de Saint-Etienne, France) and for the *C. difficile* murine model at PharmAnima platform UMS 3612 CNRS – US25 INSERM. Both platforms are conventional animal facilities with infectious sector P2. All mice were acclimated to laboratory conditions for a minimum of 1 week prior to study initiation.

### *Salmonella enterica* serovar Typhimurium infection mice model

Six- to eight-week-old female BalbC/J mice (Charles River Laboratories, Wilmington, MA, USA) individually identified by ear punches were randomly assigned to groups and housed in groups of four mice per cage and maintained on a 12-h light-dark cycle. The food (standard chow) and water were *ad libitum*. Animal viability and behavior were observed daily. Pretreatment with vancomycin (0.05 mg/g of body weight) was done on Day −5 and Day −4. One hundred microliters of treatment with pool or single donor products or treatment vehicle (PBS) was administered on Day −1 via oral gavage. Streptomycin-resistant *Salmonella enterica* serovar Typhimurium (SET) strain SL1344 inoculum (100 µL, 10^7^ CFU) was administered on Day 0 via oral gavage. Fecal sample collection occurred on Days –5, –1, 0, 3, 5. Animals were euthanized on Day 5. Animal viability and behavior were monitored daily, and survival and body weight were assessed at each time point. Mice monitoring was performed on days D-5, D-4, D-1, D0, D1, D2, D3, and D5, and weight loss was calculated from D0 as follows: % weight loss = (measured weight × 100)/ initial weight (Day 0). The disease activity index (DAI), which allows us to score the severity of colitis, was monitored daily to evaluate the clinical progression of colitis. The DAI score is cumulative and based on the presence and severity of three symptoms: weight loss, stool consistency, and rectal bleeding (Table 1). The experimental endpoint was reached when mice exhibited weight loss greater than 20% of initial weight with dehydration and diarrhea. At that point, mice were euthanized by inhalation of isoflurane followed by cervical dislocation.

**TABLE 1:** Disease activity index of *Salmonella* mouse model

Symptom	Definition	Score
Weight loss	No weight loss	0
	1%–5% loss	1
	5%–10% loss	2
	10%–20% loss	3
	>20% loss	4
Stool consistency	Normal	0
	Loose stool	2
	Diarrhea	4
Rectal bleeding	No blood	0
	Visual pellet bleeding	2
	Gross bleeding and blood around the anus	4

### *Clostridioides difficile* infection mice model

Four- to five-week-old female C57BL/6J mice (Charles River, France) individually identified by paws tattoo were randomly assigned to groups and housed in groups of three to five mice per cage and maintained under a 12-h light-dark cycle. The food (standard chow), water, bedding, and cages were autoclaved. Cage changes and daily assessment of the physical condition and behavior of the animals were performed under a laminar flow hood. All animals were treated for 3 days (D −5 to D −3) with a mixture of antibiotics (ATB) dissolved in drinking water and consisting of amikacin (0.4 mg/mL), gentamicin (0.035 mg/mL), colistin (850 U/mL), metronidazole (0.215 mg/mL), and vancomycin (0.045 mg/mL). After 1 day of wash out, mice received a single intraperitoneal injection of clindamycin (10 mg/kg of body weight) (39). The day after, mice were orally infected with 1 × 10^5^ *C. difficile* (CD) spores (R20291 strain) suspended in 200 µL of PBS for positive control group (*n* = 6) or in 200 µL of diluted (1/4 in PBS) pool or single donor products according to group assignment (*n* = 6 per group). All animals were monitored daily from infection until sacrifice for symptoms and mortality, and weights were recorded. The DAI, which allows us to score the severity of infection, was monitored daily to evaluate the progression of infection. The DAI score is cumulative and based on the presence and severity of two symptoms: weight loss and behavior with a score from 0 to 3 (Table 2). Fecal samples were collected on D −6 (before ATB treatment), D0 (before infection), and from D1 to D4 (during treatment). Animals were euthanized at D4 or before for the animals that were considered to be moribund (≥20% weight loss or DAI > 3). Mice monitoring was performed from D −6 to D4. Weight loss after D0 was calculated as follows: % weight loss=[weight (Dx) × 100]/weight (D0). The experimental endpoint was reached when mice exhibited weight loss ≥20% of initial weight or DAI > 3. Mice were euthanized by an intraperitoneal anesthesia (mix of 10 mg xylazine/kg of body weight and 150 mg ketamine/kg of body weight) followed by cervical dislocation. Survival parameter encompasses natural death and euthanasia due to a moribund state.

**TABLE 2:** Disease activity index of CDI mouse model

Score	Behavior	Weight
0	Normal	No weight loss
1	Decrease in grooming	Weight loss < 10%
2	Decrease in mobility	10% ≤ weight loss < 20%
3	Prostration	Weight loss ≥ 20%

### CFU assay

At the time of necropsy, cecal content and feces were collected, diluted in a 10 to 10 serial dilution, and plated on ChromID Petri dishes (Biomérieux) and LB (LB Broth, Sigma) supplemented with 90 µg/mL of streptomycin for *C. difficile* and *S. enterica* serovar Typhimurium titration, respectively, to quantify colony-forming units for each mouse.

### Myeloperoxidase assay

The colonic tissue section was collected and stored at −80°C until analysis. Proteins were extracted in 1 mL of PBS 1× in the presence of protease inhibitors (Roche) and ceramic balls with three cycles (30 s) of mechanical lysis. Colonic myeloperoxidase (MPO) was measured as an inflammatory marker using the Mouse MPO DuoSet Kit (R&D system) according to the manufacturer’s instructions.

### DNA isolation, 16S rDNA, and shotgun sequencing

Genomic DNA was extracted from fecal samples using the NucleoSpin Soil kit (Macherey Nagel). A sequencing library targeting the V3-V4 region of the 16S rRNA gene was constructed for each sample using the MyTaq HS-Mix (Bioline) according to the manufacturer’s instructions. Libraries were then sequenced in paired-end (2 × 300 bp) MiSeq runs (Illumina). Positive and negative controls were added throughout the process to validate the successful completion of each step.

### Bioinformatic analysis

16S rDNA bioinformatic analysis was performed with the gutPrint platform using the in-house MgTagRunner v2.0.0 pipeline. Briefly, after amplicon merging using FLASH (minimum overlap = 50 bp, maximum overlap = 300 bp, maximum 25% mismatches in the overlap region) (40), reads were quality filtered using Trimmomatic (minimum amplicon length = 300 bp, minimum average quality = 30, minimum quality for extremities = 20, and minimum quality for 10 bp sliding window = 25) (41). Amplicons were then clustered into operational taxonomic units (OTUs) with an identity threshold of 97%, and a taxonomical annotation was assigned to each output using VSEARCH (42) and the Silva SSU database (Release 128). To allow data comparison, the number of sequences was normalized to 60,000 amplicons per sample. The alpha-diversity (richness and Shannon) and beta-diversity (Jaccard and Bray-Curtis similarities) indices were calculated with R Statistical Software (R Core Team 2018, version 3.4.4) using vegan (https://cran.r-project.org/web/packages/vegan/index.html) and phyloseq packages (43).

### *In vitro* growth inhibition assay

Test samples were thawed at 37°C for 15 min in anaerobic conditions. A volume of 20 µL of each sample was added to the disks on Wilkins-Chalgren agar plates and incubated at 37°C overnight in anaerobic conditions. Blood Columbia agar and Tryptone-Soy (TS) agar were prepared according to the manufacturer’s instructions. Each targeted bacterial strain was cultured at its optimal growth media before testing (Table 3). For each bacteria preparation, colonies of each strain were added in sterile physiologic serum (NaCl 0.9%) to obtain a suspension adjusted to a visual turbidity of 1 MacFarland. One milliliter of the suspension was added to 20 mL of supercooling Mueller-Hinton agar except for *C. difficile* that was added to Brucella agar. The inoculated mixtures were poured on the first WCB layer containing disks. After solidification, an antibiotic disk specific for each strain was added, and plates were then incubated in their optimal culture condition (Table 3). The pathogen growth inhibition zone was measured manually after 24 h, and the results were expressed as the ratio of the inhibition zone of the product to that of the reference antibiotic (44). Results show the mean of one or two independent experiments preformed in triplicate. A total of 5 pooled products and 35 corresponding single donor-derived products were tested.

**TABLE 3:** Optimal culture conditions of bacteria for *in vitro* test

Pathogen species	Provider	Culture conditions before the test	Culture conditions for the test
*Clostridioides difficile* 027 R20291 strain	APHP Saint Antoine	Blood Columbia agar, anaerobic condition, 37°C, 48 h	Brucella agar, anaerobic condition, 37°C, 24 h
*Enterococcus faecium* vanA strain	APHP Saint Antoine	TS agar, aerobic condition, 37°C, 24 h	Mueller-Hinton agar, anaerobic condition, 37°C, 24 h
*Klebsiella pneumoniae* oxa48 strain	APHP Saint Antoine	TS agar, aerobic condition, 37°C, 24 h	Mueller-Hinton agar, anaerobic condition, 37°C, 24 h

### Statistical analysis

Statistics were performed using GraphPad Prism software (version 9). Unpaired *t*-tests were used for both murine and human microbiome products’ analyses. Mann-Whitney test was used for *in vitro* inhibition assays. A chi-square test was used for CD mouse survival analysis, and a two-way ANOVA followed by Tukey’s tests was used for CD and SET models.

## RESULTS

### Pooled products display reduced taxonomic variability and increased bacterial diversity compared to single donor-derived products

**Fig 1:**
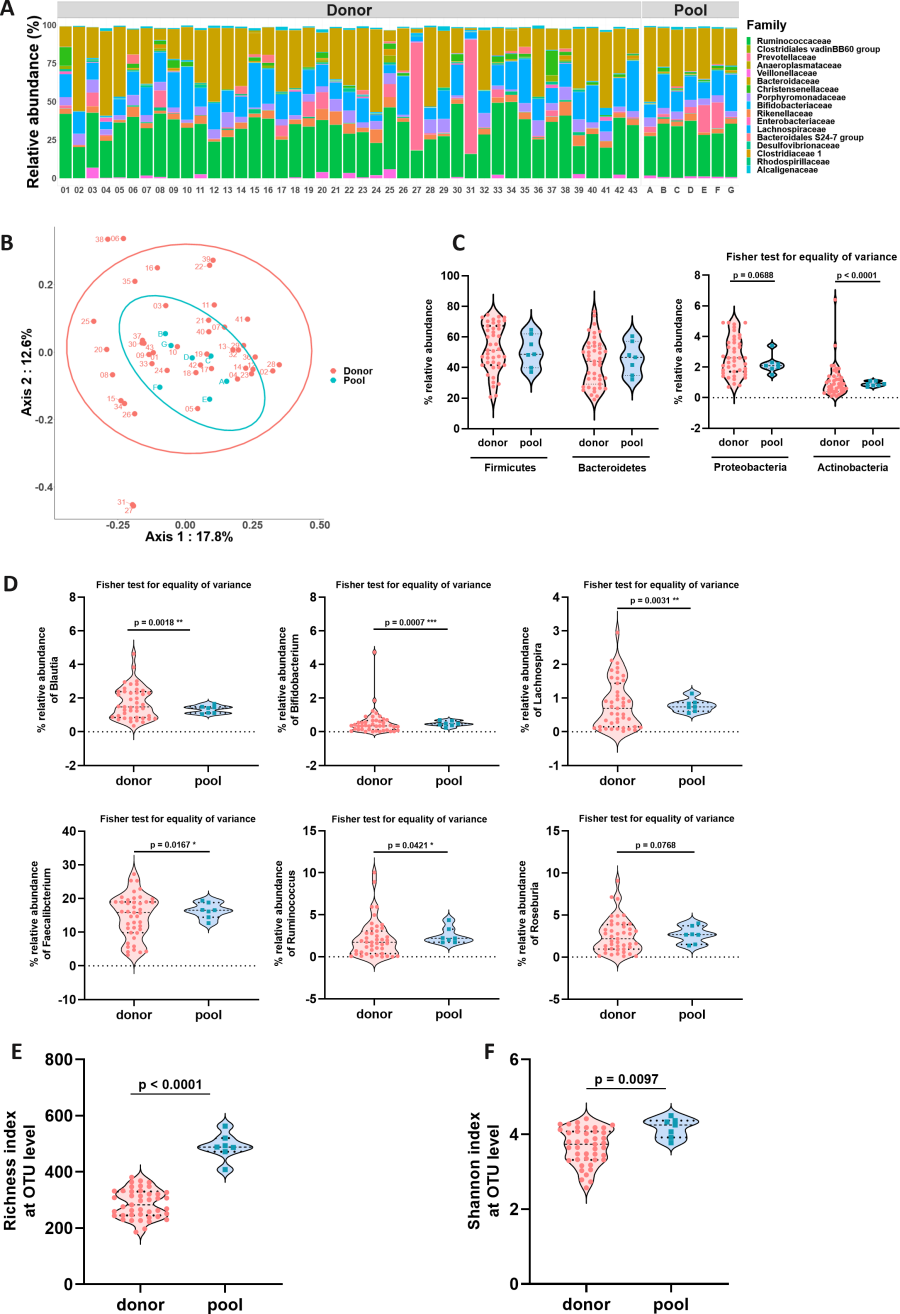
Human products’ characteristics. (**A**) Taxonomic composition at the family level. For each sample, the top abundant families covering 95% of amplicon counts were selected. Taken together, 17 families are represented. (**B**) Bray-Curtis beta-diversity at the OTU level represented by PCoA multiple distance metrics. Ellipses were drawn based on a multivariate *t*-distribution test at the 95% confidence interval for each group. Axis values correspond to the first two principal components. (**C**) Relative abundance of the four major phyla. (**D**) Relative abundance of selected bacteria associated with health benefits. (**E**) Alpha-diversity represented by OTU richness and (**F**) Shannon index at the OTU level. Unpaired *t* test was performed on richness and Shannon indexes, and Fisher’s test for equality of variance was performed on relative abundance data.

We first analyzed and compared the microbiome of all human pooled and corresponding single donor-derived products used in this study using 16S rDNA sequencing. A high taxonomic variability was observed between feces from single donors, as visually evidenced by donors 27 and 31 displaying a high relative abundance of *Prevotellaceae* compared to others (Fig. 1A). In contrast, pooled products displayed similar profiles, even when the individual donors who contributed to each pool were very different. The similarity between pooled products was evaluated using the Bray-Curtis similarity index (beta-diversity index). Bray-Curtis similarities at OTU level between pooled products were significantly higher than similarities between single donors (medians: 0.6309 vs 0.3871, respectively, *P* < 0.0001) with lower variation (Fisher’s *F* test for equality of variance, *P* = 0.0008) (Fig. S1). This was further evidenced by Bray-Curtis representation as principal coordinate analysis (PCoA), a data projection of microbial composition differences between samples. PCoA shows that the pooled products form a tight cluster of points, while individual donors are scattered (Fig. 1B), demonstrating a greater similarity between pooled product compositions. We then focused on major phyla (Fig. 1C) as well as a set of bacterial genera (*Blautia, Bifidobacterium, Lachnospira, Faecalibacterium, Ruminococcus,* and *Roseburia*) (Fig. 1D) found in healthy donors and known to be associated with health benefits. While no significant difference in their relative abundance was observed between pooled products and corresponding single donor-derived products, we observed a significant reduction in the variances of pooled products’ composition, as evidenced by Fisher’s *F* test for equality of variance. Next, we assessed the microbial richness at the OTU level (Fig. 1E), as well as the Shannon alpha-diversity index (Fig. 1F), which is representative of both the number and distribution of bacterial species. By doing so, we observed a significant increase in both the richness and Shannon diversity of pooled products compared to corresponding single donor-derived products. These results confirm that the pooling of feces from different and heterogeneous donors allows for an increase in the diversity of microbial communities while decreasing taxonomic variability between batches, as evidenced by the standardization of health-associated bacterial genera levels (18).

### Pooled microbiotherapy decreases *Salmonella enterica* serovar Typhimurium pathogenicity in mice compared to single donor-derived feces

**Fig 2:**
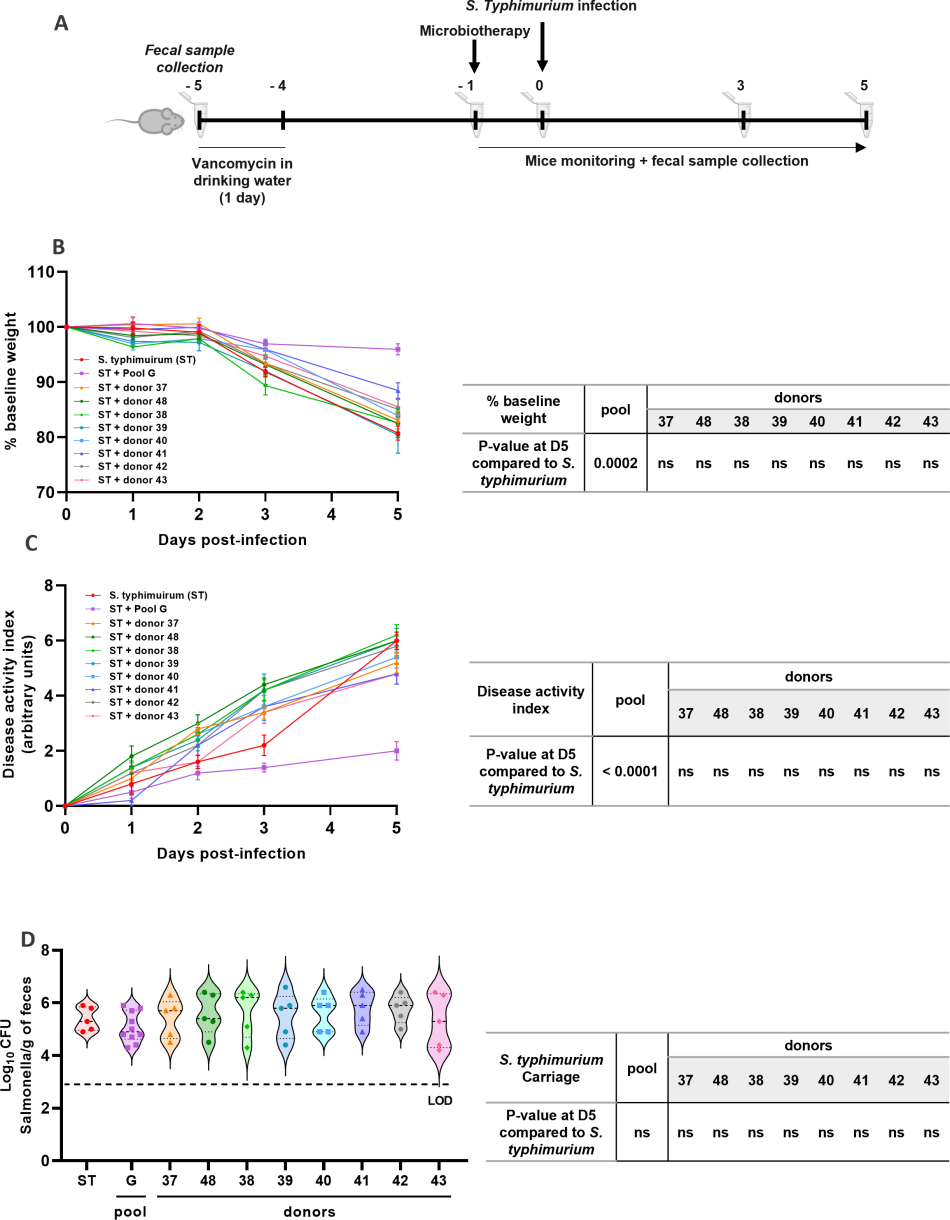
Health and biological parameters in *Salmonella* Typhimurium murine model. (**A**) Experimental design. (**B**) Monitoring of body weight. (**C**) Evolution of DAI in all groups up to the day of necropsy. (**D**) *Salmonella* Typhimurium carriage in individual mice at day 5 post-infection. LOD, limit of detection. Results are expressed as the mean ± SEM for body weight and DAI; median and range for *S.* Typhimurium carriage. Two-way ANOVA followed by *post hoc* Tukey’s tests was performed.

Next, we assessed the potential benefit of a diverse pooled microbiotherapy over corresponding single donor-derived products to protect against SET infection in mice. SET is a foodborne pathogen involved in sepsis in immunocompromised patients (45, 46) and is commonly used in mice for bacterial pathogenesis research (47). Briefly, pre-treatment with vancomycin was done on day −5 to −4 to prepare the ecological niche. Mice were then gavaged with microbiotherapy products on day −1, one day before SET infection (Fig. 2A). No mortality was reported during the experiment. High weight losses were observed in *Salmonella* group (−19.3% ± 2.9%) and in all groups treated with single donor-derived products (−19.3% ± 3.6% for donor 41; −17.5% ± 4.4% for donor 42; −17.4% ± 6.1% for donor 43; −19.6% ± 7.4% for donor 44; −16.1% ± 3.4% for donor 45; −11.5% ± 3.2% for donor 46; −14.9% ± 4.0% for donor 47; and −14.5% ± 3.3% for donor 48) 5 days post-infection (Fig. 2B). Interestingly, only a slight weight loss (−4.2% ± 3.2% for pool G) was observed in mice receiving pooled microbiotherapy at day 5 post-infection. Indeed, while no significant difference was observed between single donor-derived products and *Salmonella* group, pooled product significantly decreased weight loss (*P* = 0.0002). We further evaluated the DAI, a cumulative score based on the presence and severity of three symptoms: weight loss, stool consistency, and rectal bleeding, expressed in arbitrary units (A.U.). While pooled microbiotherapy significantly decreased the DAI (from 6.0 ± 0.7 A.U. for *Salmonella* group to 2.0 ± 1.1 A.U. for pool G; *P* < 0.0001) 5 days post-infection, no significant differences were observed between *Salmonella* group and mice treated with single donor-derived products (5.2 ± 0.8 A.U. for donor 41; 6.0 ± 0.7 A.U. for donor 42; 6.2 ± 0.8 A.U. for donor 43; 6.0 ± 1.0 A.U. for donor 44; 5.4 ± 0.9 A.U. for donor 45; 4.8 ± 0.8 A.U. for donor 46; 5.8 ± 0.8 A.U. for donor 47; and 4.8 ± 0.8 A.U. for donor 48) (Fig. 2C). Five days after infection, the feces of mice were collected and cultured to assess *Salmonella* fecal carriage as a control of infection. No clear difference in *Salmonella* fecal carriage was observed between groups 5 days post-infection (Fig. 2D). These results demonstrate the superiority of pooled microbiotherapy over corresponding single donor-derived products to decrease *Salmonella* Typhimurium pathogenicity in mice.

### Pooled microbiotherapy decreases *Clostridioides difficile* pathogenicity in mice compared to single donor-derived feces

**Fig 3:**
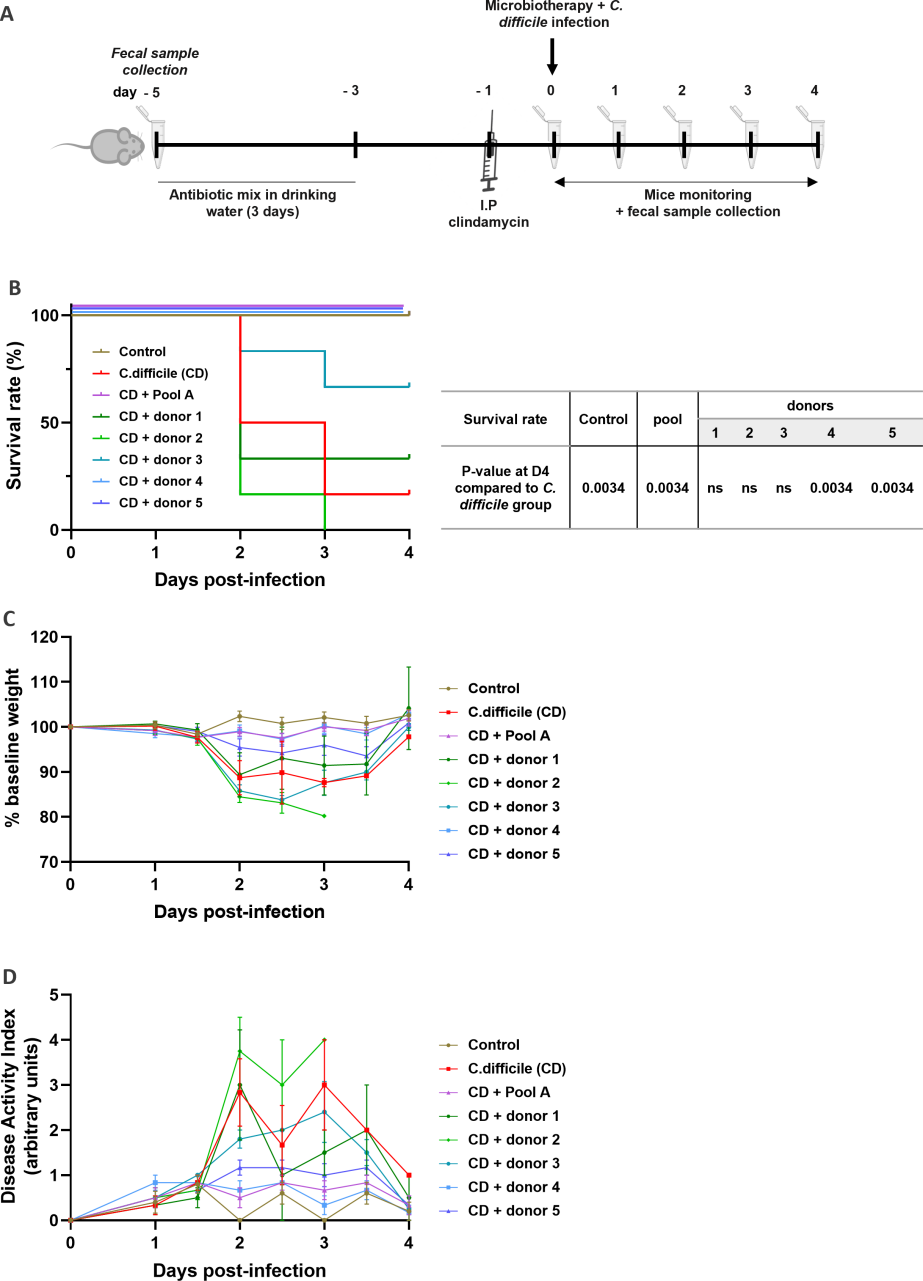
Health parameters in *C. difficile* murine model. (**A**) Experimental design. (**B**) Survival curves. (**C**) Monitoring of body weight and (**D**) DAI of all surviving mice. Results are expressed as the mean ± SEM. Chi-square test was performed on survival results.

We further compared the potential of pooled and corresponding single donor-derived microbiotherapies to prevent *Clostridioides difficile* infection in a spiking mice model. CD infection is a common intestinal infection that leads to potentially severe colitis in immune-compromised patients or in patients experiencing intensive antibiotherapies (39, 48). Briefly, mice were treated with antibiotics for 3 days to prepare the ecological niche before concomitant administration of CD and microbiotherapies (Fig. 3A). Five non-infected mice were used as negative controls (control group), and they showed a 100% survival rate. The results showed that CD challenge was highly pathogenic, resulting in a 83.3% (five out of six) mortality rate in mice (*C. difficile* group) (Fig. 3B) associated with a high body weight loss (−11.3% ± 9.4%) at 2.5 days post-infection (Fig. 3C) as previously described (39, 49, 50). We also evaluated the DAI, a cumulative score based this time on the presence and severity of two symptoms: weight loss and behavior. As expected, CD challenge resulted in a high DAI score (2.8 ± 1.8 A.U.) at 2.5 days post-infection (Fig. 3D). Strikingly, CD-infected mice treated with pooled microbiotherapy achieved 100% survival rate (Fig. 3B). This was associated with the prevention of weight loss (−1.0% ± 2.4%) and a decreased DAI score (0.5 ± 0.5 A.U.) compared to the CD group (Fig. 3C and D). On the opposite, single donor-derived products gave heterogeneous results. Indeed, the product from donor 2 failed to protect mice against CD infection, with 100% of mortality associated with a high body weight loss (−15.5% ± 2.5%) and a high DAI score (3.8 ± 1.5 A.U.) at 2.5 days post-infection. Products from donor 1 and 3 only showed moderate protective effects, with mortality rates of 66.7% and 33.3%, respectively, high body weight losses (−10.6% ± 9.8% for donor 1 and −14.2% ± 3.0% for donor 3), and high DAI scores (3.0 ± 2.4 A.U. for donor 1 and 1.8 ± 0.4 A.U. for donor 3). Finally, products from donors 4 and 5 provided strong protection against CD infection with a 100% survival rate, low body weight losses (−0.9% ± 3.3% for donor 4 and −4.5% ± 4.8% for donor 5), and low DAI scores (0.7 ± 0.5 A.U. for donor 4 and 1.2 ± 0.4 A.U. for donor 5). These results demonstrate that a pooled product is able to successfully decrease CD pathogenicity in mice while the efficacy of corresponding single donor-derived products is heterogeneous.

### Pooled microbiotherapy decreases *Clostridioides difficile* inflammation in mice compared to single donor-derived feces

**Fig 4:**
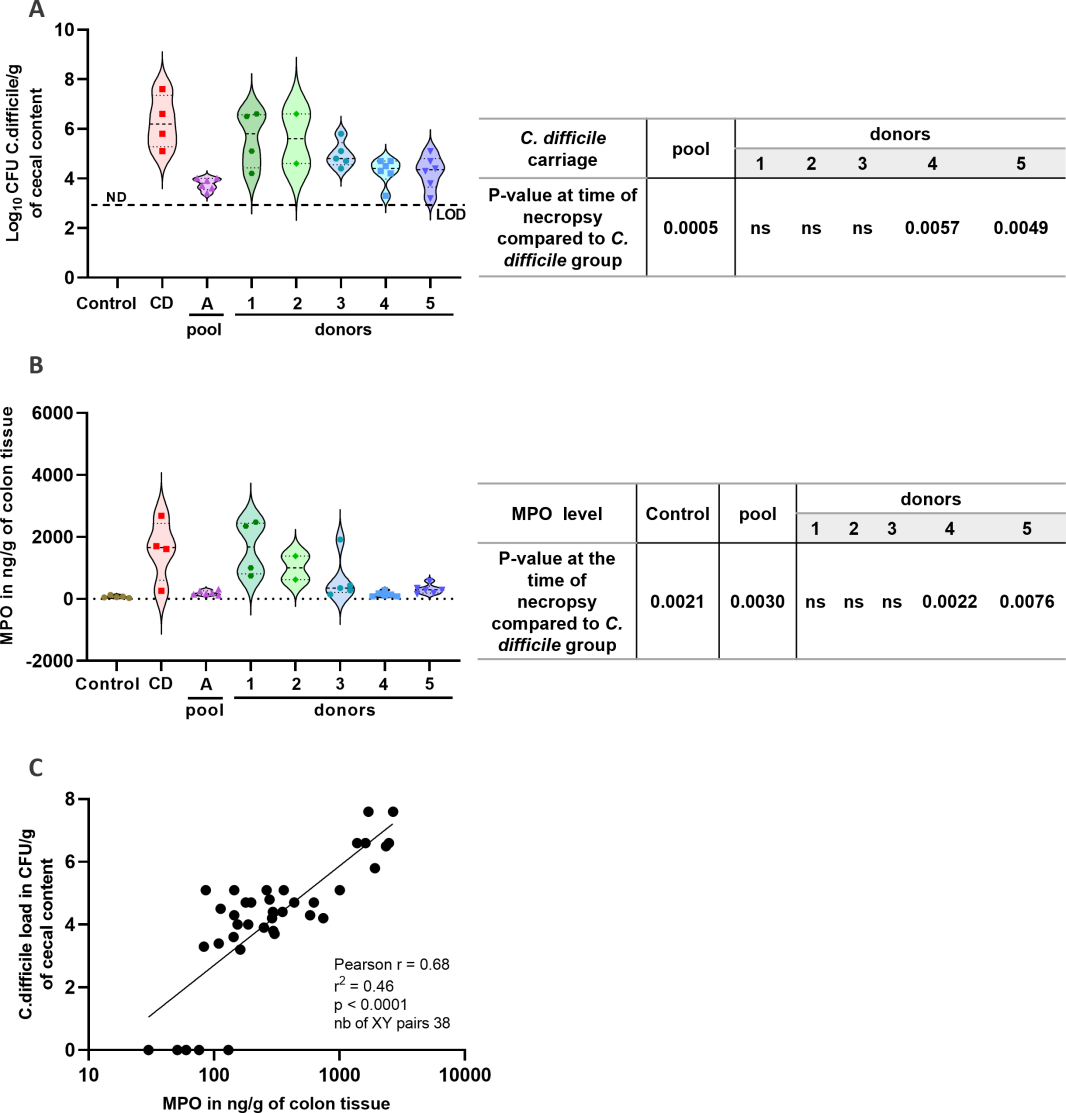
Biological parameters in *C. difficile* murine model. (**A**) Level of CD in mice cecal content at the time of necropsy. (**B**) Level of MPO in colonic tissue at the time of necropsy. (**C**) Pearson correlation analysis between *C. difficile* load in cecal content and MPO in colon. Results are expressed as the median range. ND, non-detectable. Two-way ANOVA followed by *post hoc* Tukey’s tests was performed.

CD load in the cecal content of mice treated with pooled microbiotherapy (log 3.8 ± 0.1 CFU/g; *P* = 0.0005) or with products derived from donors 4 or 5 (log 4.3 ± 0.2 CFU/g; *P* = 0.0057 and log 4.3 ± 0.3 CFU/g; *P* = 0.0049, respectively) was also significantly reduced, with values close to the limit of detection (LOD: log 3 CFU/g) compared to the CD control group (log 6.3 ± 0.5 CFU/g) (Fig. 4A). Colonic MPO, an enzyme released by neutrophils recruited to the intestinal mucosa during bacterial infections (51) and hallmark of colon inflammation, was also measured at the time of the necropsy. Two-way ANOVA revealed a significantly higher MPO level in CD-infected mice (median of 1,656 ng/g of colonic tissue) and in mice receiving donor 1-derived product (median of 1,677 ng/g of colonic tissue) compared to the control group (60 ng/g of colonic tissue; *P* < 0.005) (Fig. 4B). Mice treated with pooled microbiotherapy or with products from single donors 4 and 5 showed significantly lower colonic inflammation, with median MPO levels at least five times below those of the CD group (<300 ng/g; *P* < 0.005), in line with the CDI model description in the literature (44, 52). A significant positive correlation between CD load and MPO level was observed in mice (*P* < 0.0001, Fig. 4C). These results demonstrate that a pooled product is able to successfully decrease CD inflammation in mice while the efficacy of corresponding single donor-derived products is heterogeneous.

### Pooled microbiotherapy increases microbial diversity in *Clostridioides difficile* mice compared to single donor-derived feces

**Fig 5:**
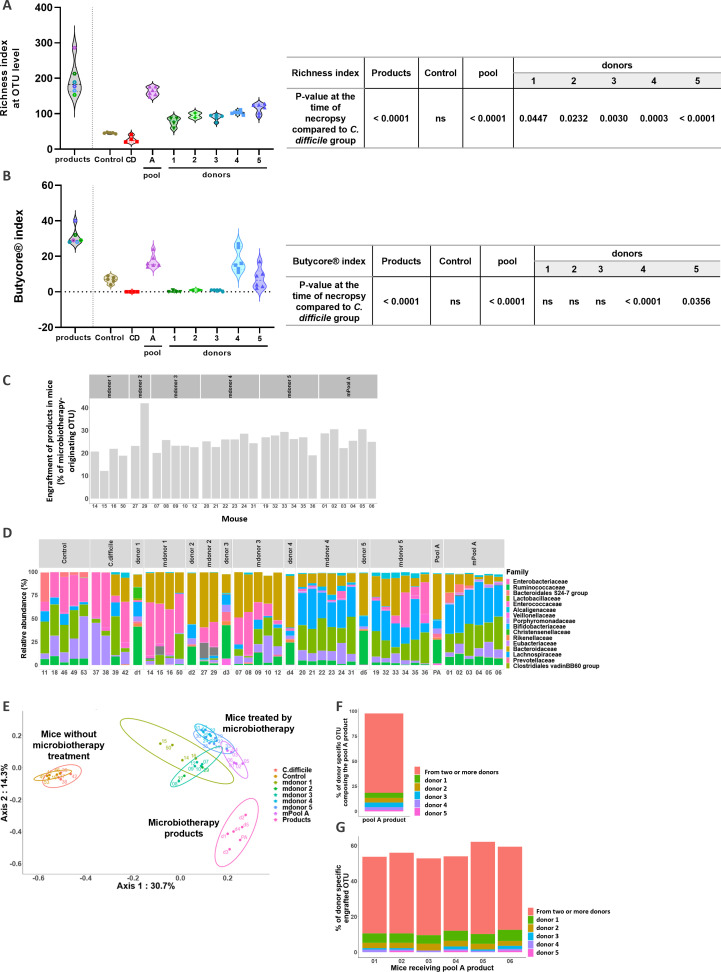
Microbiome analysis in *C. difficile* murine model at the time of necropsy. (**A**) Alpha-diversity represented by OTU richness. (**B**) Butycore index. (**C**) Engraftment of products in mice defined by the proportion of OTUs from the product found in mice microbiota after treatment. (**D**) Taxonomic composition at the family level and (**E**) Jaccard beta-diversity at OTUs’ level represented by PCoA multiple distance metrics. Axis values correspond to the first two principal components. Ellipses were drawn based on a multivariate *t*-distribution test at the 95% confidence interval for each group. (**F**) Contribution of the different donors to the pooled product expressed in percentage of OTU. (**G**) Contribution of the different donors forming the pooled product to its engraftment in mice. Two-way ANOVA followed by *post hoc* Tukey’s tests was performed. In panels **A** and **B**, empty symbols were used for products, and the same colors were conserved for mice receiving these products.

To study the changes in gut microbiome composition after CD infection and microbiotherapy, fecal samples were collected at the time of necropsy and analyzed using 16S rDNA sequencing. Treatment with both single donor-derived products and corresponding pooled products increased OTU microbial richness in mice (*P* < 0.0001) (Fig. 5A). Microbial richness was higher in mice treated with pooled products compared to mice treated with corresponding single donor-derived products (pool A: 162.8 ± 4.9 OTU; donor 1: 76.75 ± 6.7 OTU; donor 2: 95.50 ± 6.5 OTU; donor 3: 89.60 ± 3.9 OTU; donor 4: 103.0 ± 2.0 OTU; and donor 5: 114.8 ± 5.8 OTU). We then measured the Butycore index, defined as the cumulative relative abundance of 15 specific bacterial genera associated with clinical benefits and known to produce butyrate or enhance butyrate producers: *Blautia, Faecalibacterium, Alistipes, Eubacterium, Bifidobacterium, Ruminococcus, Clostridium, Coprococcus, Odoribacter, Roseburia*, *Anaerostipes, Oscillibacter, Subdoligranulum, Butyrivibrio,* and *Holdemanella*. Interestingly, CD infection induced a non-significant decrease in Butycore index in mice (6.8% ± 0.7% for control and 0.0% ± 0.0% for *C. difficile*; *P* = 0.1987) (Fig. 5B). While treatment with single donor-derived products 1, 2, and 3 failed to restore Butycore (0.25% ± 0.25% for donor 1; 1.0% ± 0.0% for donor 2; and 0.8% ± 0.2% for donor 3), pooled products as well as single donor-derived products 4 and 5 increased Butycore in treated mice (17.17% ± 1.5% for pool A; 17.83% ± 2.7% for donor 4; and 7.5% ± 2.3% for donor 5) (*P* < 0.0001, *P* < 0.0001, and *P* = 0.0356, respectively). A higher Proteobacteria/Firmicutes ratio, a classical hallmark of global gut dysbiosis (51), was also observed in CD-infected mice as well as in mice treated with single donors-derived products 1, 2, and 3 (Fig. S2A). The colonic inflammation status of mice evidenced by MPO level was positively correlated with Proteobacteria/Firmicutes ratio (Fig. S2B) (*P* < 0.0001). These results are consistent as a dysbiotic microbiome is often characterized by an overrepresentation of Proteobacteria at the expense of Firmicutes (51). At the opposite end, MPO level was negatively correlated with Butycore index (*P* < 0.005) (Fig. S2C) as well as OTU richness (*P* < 0.05) (Fig. S2D). Product engraftment was then measured as the proportion of OTUs from the product found in the microbiota of mice after treatment. Despite similar engraftment of all microbiotherapy products in mice (25.5% ± 4.7%) (Fig. 5C), in line with the literature (53), different taxonomic profiles were observed at the family level between products (Fig. 5D). Indeed, mice treated with pooled product as well as with single donor-derived products 4 and 5 display a high proportion of *Lachnospiraceae* and *Lactobacillaceae* and a low proportion of *Enterobacteriaceae* (Fig. 5D). As expected, Jaccard beta-diversity analysis demonstrated a clear stratification between products, untreated mice, and mice receiving microbiotherapy products (Fig. 5E). Mice receiving microbiotherapy products displayed a microbial profile closer to the products than to untreated mice. We further analyzed the proportions of each of the five single donor-derived products constituting the pooled product at the OTU level. By doing so, we found that the pooled product was composed of up to 79.2% of OTU shared by at least two different donors and 18.5% of donor-specific OTU in the following proportions: 5.1% from donor 1; 4.6% from donor 2; 4.6% from donor 3; 2.8% from donor 4; and 1.4% from donor 5 (Fig. 5F). Finally, we analyzed the microbiome of mice treated with pooled microbiotherapy to evaluate the percentage of donor-specific OTU engrafted in mice. These results allowed to decipher the contribution of each of the single donor-derived products composing the pool to product engraftment in mice. We found that about 45.4% ± 2.6% of the OTU engrafted in mice originated from at least two donors. Single donor-specific OTUs were found in varying proportions in mice treated with pooled microbiotherapy: 5.5% ± 0.3% from donor 1; 3.2% ± 0.2% from donor 2; 1.1% ± 0.5% from donor 3; 1.0% ± 0.2% from donor 4; and 0.8% ± 0.1% from donor 5 (Fig. 5G). Altogether, these results demonstrate that pooled microbiotherapy allows to increase the diversity of beneficial bacteria engraftment in recipient mice and support the use of pooled microbiotherapy over corresponding single donor-derived products to decrease CD pathogenicity in mice.

### Pooled products inhibit the growth of two extremely drug-resistant pathogens *in vitro*

**Fig 6:**
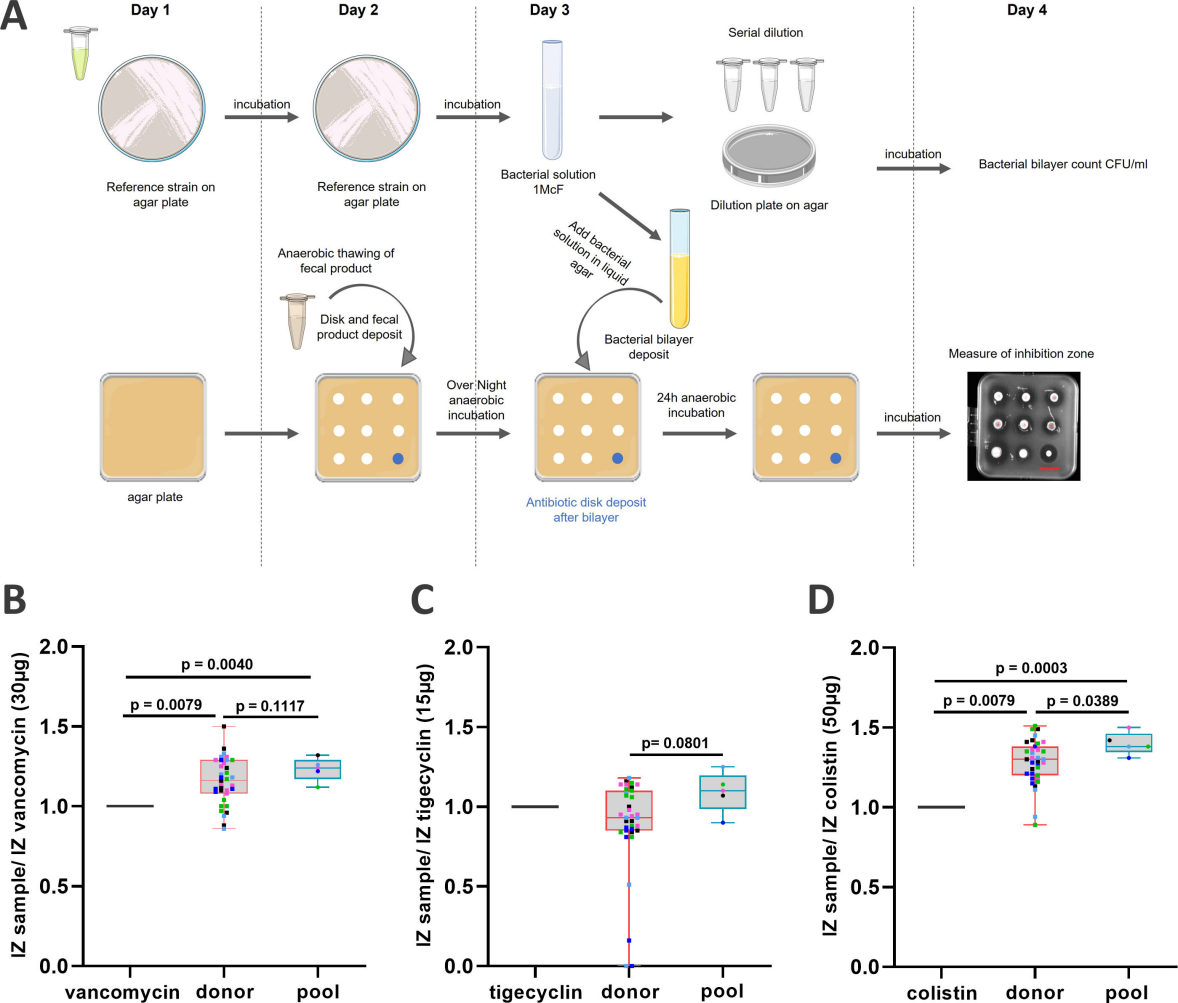
CD and eXDR antimicrobial activity of pooled and single donor products. (**A**) Experimental design, figure partially created with BioRender.com. Results of the three pathogens targeted, i.e., *C. difficile* (**B**), *E. faecium* vanA (**C**), and *K. pneumoniae* oxa48 (**D**) are expressed as the median of the ratio of inhibition zone sample/inhibition zone of reference antibiotic, i.e., vancomycin 30 µg for CD, tigecyclin 15 µg for EF, and colistin 50 µg for KP. A one-way ANOVA was performed. Single donor-derived products and corresponding pooled products share the same color code.

These *in vivo* results prompted us to compare the direct *in vitro* potential of pooled and corresponding single donor-derived microbiotherapy products to inhibit pathogen growth using a modified agar spot test assay. Briefly, after overnight incubation of the microbiotherapy samples placed on a cellulose disk, a bilayer of agar inoculated with the tested pathogen is put on top and then incubated. Pathogen growth inhibition zone was measured after 24 h, and the results were expressed as the ratio of the inhibition zone of the product to that of the reference antibiotic (Fig. 6A). First, CD pathogen growth inhibition was evaluated as the gold standard for microbiotherapy products. Interestingly, all microbiotherapy products showed anti-CD activities. While most microbiotherapy products showed anti-CD activities significantly higher compared to reference vancomycin antibiotics (*P* = 0.0079 for donor, and *P* = 0.0040 for pool) (Fig. 6B), some single donor-derived products failed to reach similar anti-microbial efficacy. These results are consistent with conclusions from the *C. difficile* infectious mouse model (Fig. 3). We further compared the anti-pathogenic activity of microbiotherapy products against *E. faecium* vanA and *K. pneumoniae* oxa48, two emerging extensively drug-resistant bacteria (eXDR) involved in sepsis in immunocompromised patients (54), with limited treatment options available (55). While most microbiotherapy products showed anti-*E*. *faecium* vanA activities (Fig. 6C), some single donor-derived products failed to demonstrate an anti-*E*. *faecium* vanA activity. The anti-pathogenic activity of pooled products was similar to that of tigecycline. A non-significant increase in anti-*E*. *faecium* vanA activity was observed with pooled products compared to single donor-derived products (*P* = 0.08). Most microbiotherapy products showed anti-*K*. *pneumoniae* oxa48 activities (Fig. 6D), which were significantly higher compared to reference colistin antibiotics (*P* = 0.0079 for donor and *P* = 0.0003 for pool). Pooled products had significantly higher *K. pneumoniae* oxa48 growth inhibition activities compared to corresponding single donor-derived products (*P* = 0.0389). Overall, pooled microbiotherapy products induced a more consistent anti-microbial activity against all three pathogens, while heterogeneous and/or lower efficacy was observed in corresponding single donor-derived products.

## DISCUSSION

The growing interest in fecal microbiotherapies (56) encounters several challenges associated with the intrinsic nature of the raw material, the challenging selection of vetted healthy donors, the need for manufacturing standardization procedures global guidance, and the lack of homogenized regulatory status between countries (26). Nonetheless, several important advances were achieved such as the emergence of stool banks (57) or the development of encapsulated freeze-dried stool formulations allowing easier storage (4°C vs −80°C) and treatment acceptability for patients (58). While significant improvements have been made, needs remain to enhance product manufacturing processes, ensuring a consistent supply of safe and effective microbiotherapies for patients. Because of the complexity of microorganism-host interactions and the diversity of fecal microbiotherapy products, elucidating the exact mechanism of action in one specific indication is challenging (33). Since its efficacy likely relies on different microbial genera or species and functions, it makes donor-dependent heterogeneity even more problematic. Thus, one of the strategies to overcome these limitations is to pool feces from several healthy donors to reduce the variability of product composition and functions, achieve higher taxonomic richness, and ensure the presence of specific bacterial genera with health benefits such as butyrate-producing bacteria.

By comparing the microbiome profile of single donors and corresponding pooled products, we demonstrated that the pooling strategy successfully reduces donor-dependent microbiome product variability, especially for bacterial genera associated with health benefits such as *Blautia* spp., *Faecalibacterium* spp., *Ruminococcus* spp., and *Bifidobacterium* spp. The Shannon alpha-diversity index, which has been used as a healthy microbiota indicator in human studies (59), is also significantly increased in pooled products. In line, in the HERACLES clinical study evaluating pooled fecal microbiotherapy in SR-GI-aGvHD patients, an increase in both richness and Shannon index was observed in pooled products and stool from responder patients after pooled microbiotherapy. The presence of a group of 15 different bacterial genera known to produce short chain fatty acid also called Butycore index was further observed in both microbiotherapy products and treated responder patients (18). Finally, Średnicka and colleagues (60) also observed a reduced variability and an increase in alpha-diversity metrics in fecal pooled inoculum used for *in vitro* culture.

Infectious diseases remain one of the main causes of mortality and morbidity in the world (61), due to the quickly escalating emergence of antibiotic resistance. Here, we evidenced for the first time the capability of a pooled microbiotherapy product to prevent the appearance of symptoms induced by SET infection, while some corresponding single donor-derived products failed to be effective. Interestingly, previous work performed by Wang and colleagues (62) also failed to demonstrate the efficacy of a single donor-derived mice product, although this discrepancy might be related to a different experimental design aiming at curing the infection. Surprisingly, the improved DAI score obtained after pooled fecal microbiotherapy was not associated with a significant decrease in fecal *S.* Typhimurium load. This could be explained by the short duration of the experiment and SET’s ability to long-term colonize the digestive tract (62).

Confirmation of the protective effect of pooled products compared to single donor-derived products was demonstrated using a spiking CD mouse infection model. CD is the first cause of hospital-acquired infections and is also involved in sepsis in immunocompromised patients. CD infection is the first indication for which a fecal microbiotherapy product has been approved by the US FDA. As such, it is now considered as the gold standard for the evaluation of fecal microbiotherapies in infectious diseases, although CD mouse model humanized with human microbiota cannot be considered an exact reflection of rCDI patients’ clinical care (63). We revealed that a pooled product was able to successfully protect against CD infection while the efficacy of corresponding single donor-derived products was heterogeneous. The microbiome profile of rCDI patients is characterized by a decrease in both bacterial diversity and richness, reflected by a drop in the relative abundance of *Ruminococcaceae, Lachnospiraceae,* and butyrate-producing bacteria belonging to cluster IV and XIVa of Clostridiales (64). Following microbiotherapy, a structural change in the intestinal microbiota of patients is observed with an increase in the relative abundance of Bacteroidetes and a decrease in Proteobacteria (65, 66). Similarly, CDI animal models are characterized by a less diversified microbial profile with a low Firmicutes/Bacteroidetes ratio and an increase in the proportion of Proteobacteria such as *Enterobacteriaceae* (67).

In this study, CD-infected mice showed a decreased microbial richness, which was recovered after microbiotherapy. Microbial richness increased to a higher level for mice treated with pooled product compared to mice treated with corresponding single donor-derived products. CDI-infected mice showed a low relative abundance of Firmicutes, in particular, *Ruminococcaceae, Lachnospiraceae,* and *Lactobacillaceae*, and a high abundance of Proteobacteria, namely *Enterobacteriaceae*. This profile, characterized by a high Proteobacteria/Firmicutes ratio, was also observed in mice receiving the least effective products from donors 1, 2, and 3 and positively correlates with colonic inflammation. Indeed, Proteobacteria, a phylum characterized by a high presence of endotoxin-bearing bacteria, is positively correlated with fecal calprotectin, an inflammation marker used in intestinal bowel disease (IBD) disease activity monitoring (68). Enrichment of bacterial genera associated with health benefits highlighted by a high Butycore index was also observed in mice treated with pooled product, while the results were heterogenous for mice treated with single donor-derived products. Among the genera found in Butycore index, some of them like *Coprococcus_2, Roseburia,* or *Ruminococcus* spp. are known to have anti-CD activity through the production of short chain fatty acids such as butyrate. Butyrate is able to alleviate intestinal inflammation via the stabilization of hypoxia-inducible factor-1 expression, preventing bacterial translocation and local inflammatory response (69). In addition, other genera used in Butycore index such as *Bifidobacterium, Dorea,* or *Ruminococcus* spp. are able to replace CD through niche competition due to their mucinophilic properties (33). Another known mechanism of action of microbiotherapy against *C. difficile* infection is the modulation of bile-salt metabolism. Indeed, while primary bile acids stimulate the germination of spores, secondary bile acids such as lithocholate and deoxycholic acid are potent inhibitors of spore germination (70). In particular, genera like *Blautia, Roseburia, Ruminococcus,* or *Bifidobacterium* spp. included in Butycore index are known to have the enzymatic ability to produce secondary bile acids (71). Nevertheless, further mechanistic investigation should be performed to support these hypotheses.

*In vitro* results further showed that single donor-derived products have very variable anti-microbial activity against CD and two emerging extensively drug-resistant bacteria, *Enterococcus faecium* vanA and *Klebsiella pneumoniae* oxa48. On the opposite end, the anti-microbial activity of corresponding pooled products seemed more consistent between batches. These results are in line with the reduction in the variability of health-benefiting bacterial genera observed in pooled products since the anti-microbial activity of bacterial strains is often attributed to the production of metabolites such as short chain fatty acids, hydrogen peroxide, ethanol, acetaldehyde, reuterin, and other bacteriocins (72). These preliminary results support the use of homogeneous and enriched pooled fecal microbiotherapies against eXDR bacteria, for which few treatment options are available and effective. Nonetheless, further investigation should be performed to endorse these results.

Altogether, both *in vivo* and *in vitro* results demonstrate that the heterogeneous efficacy of single donor-derived products to treat infectious diseases is corrected by pooled fecal microbiotherapy. Using comprehensive meta-analyses and machine learning, microbiota engraftment, donor-recipient complementarity, and mode of administration were identified as key success factors for microbiotherapy (73, 74). Especially, Ianiro et al. (74) showed that better strain engraftment was associated with improved clinical outcomes in a global analysis across 24 studies. Machine learning predictions further suggest that microbiotherapy using high-richness donors should yield higher microbial richness in severe dysbiotic recipient patients. These results are consistent with previous works highlighting the association between microbiotherapy richness and efficacy (28, 30, 75). In this study, we demonstrate that mice treated with pooled microbiotherapy were colonized by common OTU shared by several donors but also by specific OTU originating from each donor (composing the pooled product). These results indicate that pooled microbiotherapy allows to increase the diversity of beneficial bacterial engraftment in recipient mice.

Thus, these data support the use of high-richness pooled microbiotherapies to increase the likelihood of donor-recipient matching. This study also supports promising clinical results obtained using pooled microbiotherapy in different indications such as obesity (75), rCDI (76), UC (32, 77–79), and SR-GI-aGvHD (18). In line, mathematical modeling suggests that the pooling of stools—via daily cycling of encapsulated stool from several healthy donors—may be beneficial for FMT treatment of chronic microbiota-related diseases (31). Finally, recent preclinical data demonstrate that pooled microbiotherapies are more effective than single donor-derived products to alleviate the symptoms of high-fat-diet-induced hepatic steatosis in mice (80). Altogether, these data support the development of high richness and diversity-pooled microbiotherapies capable of overcoming the recipient effect, making them adapted to a higher number of patients.

## Data Availability

Sequencing data that support the findings have been deposited in the NCBI SRA database under BioProject accession number PRJNA1086865.

## SUPPLEMENTAL MATERIAL

Supplemental tables and figures aem.00016-24-s0001.docx Table S1; Figures S1 and S2.

ASM does not own the copyrights to Supplemental Material that may be linked to, or accessed through, an article. The authors have granted ASM a non-exclusive, world-wide license to publish the Supplemental Material files. Please contact the corresponding author directly for reuse.

## References

[1] Thursby E, Juge N. 2017. Introduction to the human gut microbiota. Biochem J 474:1823–1836. doi:10.1042/BCJ20160510 28512250 PMC5433529

[2] van Nood E, Vrieze A, Nieuwdorp M, Fuentes S, Zoetendal EG, de Vos WM, Visser CE, Kuijper EJ, Bartelsman J, Tijssen JGP, Speelman P, Dijkgraaf MGW, Keller JJ. 2013. Duodenal infusion of donor feces for recurrent Clostridium difficile. N Engl J Med 368:407–415. doi:10.1056/NEJMoa1205037 23323867

[3] Kelly CR, Khoruts A, Staley C, Sadowsky MJ, Abd M, Alani M, Bakow B, Curran P, McKenney J, Tisch A, Reinert SE, Machan JT, Brandt LJ. 2016. Effect of fecal microbiota transplantation on recurrence in multiply recurrent Clostridium difficile infection: a randomized trial. Ann Intern Med 165:609–616. doi:10.7326/M16-0271 27547925 PMC5909820

[4] Lee CH, Steiner T, Petrof EO, Smieja M, Roscoe D, Nematallah A, Weese JS, Collins S, Moayyedi P, Crowther M, Ropeleski MJ, Jayaratne P, Higgins D, Li Y, Rau NV, Kim PT. 2016. Frozen vs fresh fecal microbiota transplantation and clinical resolution of diarrhea in patients with recurrent Clostridium difficile infection: a randomized clinical trial. JAMA 315:142–149. doi:10.1001/jama.2015.18098 26757463

[5] Khanna S, Assi M, Lee C, Yoho D, Louie T, Knapple W, Aguilar H, Garcia-Diaz J, Wang GP, Berry SM, Marion J, Su X, Braun T, Bancke L, Feuerstadt P. 2022. Efficacy and safety of RBX2660 in PUNCH CD3, a phase III, randomized, double-blind, placebo-controlled trial with a Bayesian primary analysis for the prevention of recurrent Clostridioides difficile infection. Drugs 82:1527–1538. doi:10.1007/s40265-022-01797-x 36287379 PMC9607700

[6] Boicean A, Neamtu B, Birsan S, Batar F, Tanasescu C, Dura H, Roman MD, Hașegan A, Bratu D, Mihetiu A, Mohor CI, Mohor C, Bacila C, Negrea MO, Fleaca SR. 2022. Fecal microbiota transplantation in patients co-infected with SARS-CoV2 and Clostridioides difficile. Biomedicines 11:7. doi:10.3390/biomedicines11010007 36672518 PMC9855959

[7] Kuijper EJ, Vendrik KEW, Vehreschild M. 2019. Manipulation of the microbiota to eradicate multidrug-resistant Enterobacteriaceae from the human intestinal tract. Clin Microbiol Infect 25:786–789. doi:10.1016/j.cmi.2019.03.025 30965098

[8] Serrano-Villar S, Talavera-Rodríguez A, Gosalbes MJ, Madrid N, Pérez-Molina JA, Elliott RJ, Navia B, Lanza VF, Vallejo A, Osman M, Dronda F, Budree S, Zamora J, Gutiérrez C, Manzano M, Vivancos MJ, Ron R, Martínez-Sanz J, Herrera S, Ansa U, Moya A, Moreno S. 2021. Fecal microbiota transplantation in HIV: a pilot placebo-controlled study. Nat Commun 12:1139. doi:10.1038/s41467-021-21472-1 33602945 PMC7892558

[9] Chen Q, Fan Y, Zhang B, Yan C, Zhang Q, Ke Y, Chen Z, Wang L, Shi H, Hu Y, Huang Q, Su J, Xie C, Zhang X, Zhou L, Ren J, Xu H. 2023. Capsulized fecal microbiota transplantation induces remission in patients with ulcerative colitis by gut microbial colonization and metabolite regulation. Microbiol Spectr 11:e0415222. doi:10.1128/spectrum.04152-22 37093057 PMC10269780

[10] Cheng F, Huang Z, Wei W, Li Z. 2021. Fecal microbiota transplantation for Crohn’s disease: a systematic review and meta-analysis. Tech Coloproctol 25:495–504. doi:10.1007/s10151-020-02395-3 33759066

[11] de Groot P, Nikolic T, Pellegrini S, Sordi V, Imangaliyev S, Rampanelli E, Hanssen N, Attaye I, Bakker G, Duinkerken G, et al.. 2021. Faecal microbiota transplantation halts progression of human new-onset type 1 diabetes in a randomised controlled trial. Gut 70:92–105. doi:10.1136/gutjnl-2020-322630 33106354 PMC7788262

[12] Wang H, Lu Y, Yan Y, Tian S, Zheng D, Leng D, Wang C, Jiao J, Wang Z, Bai Y. 2019. Promising treatment for type 2 diabetes: fecal microbiota transplantation reverses insulin resistance and impaired islets. Front Cell Infect Microbiol 9:455. doi:10.3389/fcimb.2019.00455 32010641 PMC6979041

[13] Kragsnaes MS, Kjeldsen J, Horn HC, Munk HL, Pedersen JK, Just SA, Ahlquist P, Pedersen FM, de Wit M, Möller S, Andersen V, Kristiansen K, Kinggaard Holm D, Holt HM, Christensen R, Ellingsen T. 2021. Safety and efficacy of faecal microbiota transplantation for active peripheral psoriatic arthritis: an exploratory randomised placebo-controlled trial. Ann Rheum Dis 80:1158–1167. doi:10.1136/annrheumdis-2020-219511 33926922

[14] Jagessar SAR, Long C, Cui B, Zhang F. 2019. Improvement of Good’s syndrome by fecal microbiota transplantation: the first case report. J Int Med Res 47:3408–3415. doi:10.1177/0300060519854913 31218940 PMC6683929

[15] Evrensel A, Önen Ünsalver B, Ceylan ME. 2019. Therapeutic potential of the microbiome in the treatment of neuropsychiatric disorders. Med Sci (Basel) 7:21. doi:10.3390/medsci7020021 30709065 PMC6410187

[16] Xue L, Deng Z, Luo W, He X, Chen Y. 2022. Effect of fecal microbiota transplantation on non-alcoholic fatty liver disease: a randomized clinical trial. Front Cell Infect Microbiol 12:759306. doi:10.3389/fcimb.2022.759306 35860380 PMC9289257

[17] Caggiano G, Stasi A, Franzin R, Fiorentino M, Cimmarusti MT, Deleonardis A, Palieri R, Pontrelli P, Gesualdo L. 2023. Fecal microbiota transplantation in reducing uremic toxins accumulation in kidney disease: current understanding and future perspectives. Toxins (Basel) 15:115. doi:10.3390/toxins15020115 36828429 PMC9965504

[18] Malard F, Loschi M, Huynh A, Cluzeau T, Guenounou S, Legrand F, Magro L, Orvain C, Charbonnier A, Panz-Klapuch M, et al.. 2023. Pooled allogeneic faecal microbiota MaaT013 for steroid-resistant gastrointestinal acute graft-versus-host disease: a single-arm, multicentre phase 2 trial. EClinicalMedicine 62:102111. doi:10.1016/j.eclinm.2023.102111 37654670 PMC10466244

[19] Wang Y, Jenq RR, Wargo JA, Watowich SS. 2023. Microbiome influencers of checkpoint blockade–associated toxicity. J Exp Med 220:e20220948. doi:10.1084/jem.20220948 36622383 PMC9836236

[20] Malard F, Vekhoff A, Lapusan S, Isnard F, D’incan-Corda E, Rey J, Saillard C, Thomas X, Ducastelle-Lepretre S, Paubelle E, et al.. 2021. Gut microbiota diversity after autologous fecal microbiota transfer in acute myeloid leukemia patients. Nat Commun 12:3084. doi:10.1038/s41467-021-23376-6 34035290 PMC8149453

[21] Daillère R, Derosa L, Bonvalet M, Segata N, Routy B, Gariboldi M, Budinská E, De Vries IJM, Naccarati AG, Zitvogel V, Caldas C, Engstrand L, Loilbl S, Fieschi J, Heinzerling L, Kroemer G, Zitvogel L. 2020. Trial watch: the gut microbiota as a tool to boost the clinical efficacy of anticancer immunotherapy. Oncoimmunology 9:1774298. doi:10.1080/2162402X.2020.1774298 32934879 PMC7466862

[22] McQuade JL, Ologun GO, Arora R, Wargo JA. 2020. Gut microbiome modulation via fecal microbiota transplant to augment immunotherapy in patients with melanoma or other cancers. Curr Oncol Rep 22:74. doi:10.1007/s11912-020-00913-y 32577835 PMC7685568

[23] Hvas CL, Keller J, Baunwall SMD, Edwards LA, Ianiro G, Kupcinskas J, Link A, Mullish BH, Satokari R, Sokol H, Terveer E, Vehreshild MJG. 2020. European academic faecal microbiota transplantation (EURFMT) network: improving the safety and quality of microbiome therapies in Europe. Microb Health Dis 5:e954. doi:10.26355/mhd_202311_954

[24] Zhang X, Ishikawa D, Nomura K, Fukuda N, Haraikawa M, Haga K, Shibuya T, Mita T, Nagahara A. 2022. Donor screening revisions of fecal microbiota transplantation in patients with ulcerative colitis. J Clin Med 11:1055. doi:10.3390/jcm11041055 35207328 PMC8879222

[25] Goldenberg SD, Batra R, Beales I, Digby-Bell JL, Irving PM, Kellingray L, Narbad A, Franslem-Elumogo N. 2018. Comparison of different strategies for providing fecal microbiota transplantation to treat patients with recurrent Clostridium difficile infection in two english hospitals: a review. Infect Dis Ther 7:71–86. doi:10.1007/s40121-018-0189-y 29450831 PMC5840108

[26] Nicco C, Paule A, Konturek P, Edeas M. 2020. From donor to patient: collection, preparation and cryopreservation of fecal samples for fecal microbiota transplantation. Diseases 8:9. doi:10.3390/diseases8020009 32326509 PMC7349373

[27] Mizuno S, Masaoka T, Naganuma M, Kishimoto T, Kitazawa M, Kurokawa S, Nakashima M, Takeshita K, Suda W, Mimura M, Hattori M, Kanai T. 2017. Bifidobacterium-rich fecal donor may be a positive predictor for successful fecal microbiota transplantation in patients with irritable bowel syndrome. Digestion 96:29–38. doi:10.1159/000471919 28628918 PMC5637308

[28] Vermeire S, Joossens M, Verbeke K, Wang J, Machiels K, Sabino J, Ferrante M, Van Assche G, Rutgeerts P, Raes J. 2016. Donor species richness determines faecal microbiota transplantation success in inflammatory bowel disease. J Crohns Colitis 10:387–394. doi:10.1093/ecco-jcc/jjv203 26519463 PMC4946755

[29] Moayyedi P, Surette MG, Kim PT, Libertucci J, Wolfe M, Onischi C, Armstrong D, Marshall JK, Kassam Z, Reinisch W, Lee CH. 2015. Fecal microbiota transplantation induces remission in patients with active ulcerative colitis in a randomized controlled trial. Gastroenterology 149:102–109. doi:10.1053/j.gastro.2015.04.001 25857665

[30] Kump P, Wurm P, Gröchenig HP, Wenzl H, Petritsch W, Halwachs B, Wagner M, Stadlbauer V, Eherer A, Hoffmann KM, Deutschmann A, Reicht G, Reiter L, Slawitsch P, Gorkiewicz G, Högenauer C. 2018. The taxonomic composition of the donor intestinal microbiota is a major factor influencing the efficacy of faecal microbiota transplantation in therapy refractory ulcerative colitis. Aliment Pharmacol Ther 47:67–77. doi:10.1111/apt.14387 29052237 PMC5765501

[31] Kazerouni A, Wein LM. 2017. Exploring the efficacy of pooled stools in fecal microbiota transplantation for microbiota-associated chronic diseases. PLoS One 12:e0163956. doi:10.1371/journal.pone.0163956 28068341 PMC5221766

[32] Paramsothy S, Kamm MA, Kaakoush NO, Walsh AJ, van den Bogaerde J, Samuel D, Leong RWL, Connor S, Ng W, Paramsothy R, Xuan W, Lin E, Mitchell HM, Borody TJ. 2017. Multidonor intensive faecal microbiota transplantation for active ulcerative colitis: a randomised placebo-controlled trial. Lancet 389:1218–1228. doi:10.1016/S0140-6736(17)30182-4 28214091

[33] Tian H, Cui J, Ye C, Zhao J, Yang B, Xu Y, Ji S, Wang L, Lv X, Ma C, Zhou S, Li N, Wang X, Qin H, Chen Q. 2023. Depletion of butyrate-producing microbes of the firmicutes predicts nonresponse to FMT therapy in patients with recurrent Clostridium difficile infection. Gut Microbes 15:2236362. doi:10.1080/19490976.2023.2236362 37469017 PMC10361143

[34] Merrick B, Allen L, Masirah M Zain N, Forbes B, Shawcross DL, Goldenberg SD. 2020. Regulation, risk and safety of faecal microbiota transplant. Infect Prev Pract 2:100069. doi:10.1016/j.infpip.2020.100069 34316559 PMC7280140

[35] DeFilipp Z, Bloom PP, Torres Soto M, Mansour MK, Sater MRA, Huntley MH, Turbett S, Chung RT, Chen Y-B, Hohmann EL. 2019. Drug-resistant E. coli bacteremia transmitted by fecal microbiota transplant. N Engl J Med 381:2043–2050. doi:10.1056/NEJMoa1910437 31665575

[36] Malard F, Loschi M, Cluzeau T, Legrand F, Mear J-B, Lhomme F, Guenounou S, Huynh A, Borel C, Desmier D, et al.. 2022. Pooled fecal allogenic microbiotherapy for refractory gastrointestinal acute graft-versus-host disease: results from the early access program in France. Blood 140:276–278. doi:10.1182/blood-2022-163340

[37] Levast B, Fontaine M, Nancey S, Dechelotte P, Doré J, Lehert P. 2023. Single-donor and pooling strategies for fecal microbiota transfer product preparation in ulcerative colitis: a systematic review and meta-analysis. Clin Transl Gastroenterol 14:e00568. doi:10.14309/ctg.0000000000000568 37232579 PMC10208705

[38] Burz SD, Abraham A-L, Fonseca F, David O, Chapron A, Béguet-Crespel F, Cénard S, Le Roux K, Patrascu O, Levenez F, Schwintner C, Blottière HM, Béra-Maillet C, Lepage P, Doré J, Juste C. 2019. A guide for ex vivo handling and storage of stool samples intended for fecal microbiota transplantation. Sci Rep 9:8897. doi:10.1038/s41598-019-45173-4 31222022 PMC6586871

[39] Chen X, Katchar K, Goldsmith JD, Nanthakumar N, Cheknis A, Gerding DN, Kelly CP. 2008. A mouse model of Clostridium difficile–associated disease. Gastroenterology 135:1984–1992. doi:10.1053/j.gastro.2008.09.002 18848941

[40] Magoč T, Salzberg SL. 2011. FLASH: fast length adjustment of short reads to improve genome assemblies. Bioinformatics 27:2957–2963. doi:10.1093/bioinformatics/btr507 21903629 PMC3198573

[41] Bolger AM, Lohse M, Usadel B. 2014. Trimmomatic: a flexible trimmer for Illumina sequence data. Bioinformatics 30:2114–2120. doi:10.1093/bioinformatics/btu170 24695404 PMC4103590

[42] Rognes T, Flouri T, Nichols B, Quince C, Mahé F. 2016. VSEARCH: a versatile open source tool for metagenomics. PeerJ 4:e2584. doi:10.7717/peerj.2584 27781170 PMC5075697

[43] McMurdie PJ, Holmes S. 2013. phyloseq: an R package for reproducible interactive analysis and graphics of microbiome census data. PLoS One 8:e61217. doi:10.1371/journal.pone.0061217 23630581 PMC3632530

[44] Reygner J, Charrueau C, Delannoy J, Mayeur C, Robert V, Cuinat C, Meylheuc T, Mauras A, Augustin J, Nicolis I, Modoux M, Joly F, Waligora-Dupriet A-J, Thomas M, Kapel N. 2020. Freeze-dried fecal samples are biologically active after long-lasting storage and suited to fecal microbiota transplantation in a preclinical murine model of Clostridioides difficile infection. Gut Microbes 11:1405–1422. doi:10.1080/19490976.2020.1759489 32501140 PMC7524285

[45] Dadwal SS, Tegtmeier B, Nakamura R, Kriengkauykiat J, Ito J, Forman SJ, Pullarkat V. 2011. Nontyphoidal Salmonella infection among recipients of hematopoietic SCT. Bone Marrow Transplant 46:880–883. doi:10.1038/bmt.2010.204 20838389

[46] Ko P-S, Liu Y-C, Wang H-Y, Wu C-Y, Fan N-W, Liu C-J, Yu Y-B, Hsiao L-T, Chiou T-J, Tzeng C-H, Liu J-H, Gau J-P. 2017. Clinical-associated characteristics and microbiological features of bloodstream nontyphoidal Salmonella infection in adult patients receiving allogeneic hematopoietic stem cell transplantation. Ann Hematol 96:1533–1540. doi:10.1007/s00277-017-3054-2 28710648

[47] Tsolis RM, Xavier MN, Santos RL, Bäumler AJ. 2011. How to become a top model: impact of animal experimentation on human Salmonella disease research. Infect Immun 79:1806–1814. doi:10.1128/IAI.01369-10 21343352 PMC3088149

[48] Jiang Z-D, Alexander A, Ke S, Valilis EM, Hu S, Li B, DuPont HL. 2017. Stability and efficacy of frozen and lyophilized fecal microbiota transplant (FMT) product in a mouse model of Clostridium difficile infection (CDI). Anaerobe 48:110–114. doi:10.1016/j.anaerobe.2017.08.003 28801119

[49] Reeves AE, Theriot CM, Bergin IL, Huffnagle GB, Schloss PD, Young VB. 2011. The interplay between microbiome dynamics and pathogen dynamics in a murine model of Clostridium difficile Infection. Gut Microbes 2:145–158. doi:10.4161/gmic.2.3.16333 21804357 PMC3225775

[50] Theriot CM, Koumpouras CC, Carlson PE, Bergin II, Aronoff DM, Young VB. 2011. Cefoperazone-treated mice as an experimental platform to assess differential virulence of Clostridium difficile strains. Gut Microbes 2:326–334. doi:10.4161/gmic.19142 22198617 PMC3337121

[51] Bäumler AJ, Sperandio V. 2016. Interactions between the microbiota and pathogenic bacteria in the gut. Nature 535:85–93. doi:10.1038/nature18849 27383983 PMC5114849

[52] Seekatz AM, Theriot CM, Molloy CT, Wozniak KL, Bergin IL, Young VB. 2015. Fecal microbiota transplantation eliminates Clostridium difficile in a murine model of relapsing disease. Infect Immun 83:3838–3846. doi:10.1128/IAI.00459-15 26169276 PMC4567621

[53] Lundberg R, Toft MF, Metzdorff SB, Hansen CHF, Licht TR, Bahl MI, Hansen AK. 2020. Human microbiota-transplanted C57BL/6 mice and offspring display reduced establishment of key bacteria and reduced immune stimulation compared to mouse microbiota-transplantation. Sci Rep 10:7805. doi:10.1038/s41598-020-64703-z 32385373 PMC7211022

[54] Garcia-Vidal C, Cardozo-Espinola C, Puerta-Alcalde P, Marco F, Tellez A, Agüero D, Romero-Santana F, Díaz-Beyá M, Giné E, Morata L, Rodríguez-Núñez O, Martinez JA, Mensa J, Esteve J, Soriano A. 2018. Risk factors for mortality in patients with acute leukemia and bloodstream infections in the era of multiresistance. PLoS One 13:e0199531. doi:10.1371/journal.pone.0199531 29953464 PMC6023133

[55] Cassini A, Högberg LD, Plachouras D, Quattrocchi A, Hoxha A, Simonsen GS, Colomb-Cotinat M, Kretzschmar ME, Devleesschauwer B, Cecchini M, Ouakrim DA, Oliveira TC, Struelens MJ, Suetens C, Monnet DL, Burden of AMR Collaborative Group. 2019. Attributable deaths and disability-adjusted life-years caused by infections with antibiotic-resistant bacteria in the EU and the European economic area in 2015: a population-level modelling analysis. Lancet Infect Dis 19:56–66. doi:10.1016/S1473-3099(18)30605-4 30409683 PMC6300481

[56] Gebrayel P, Nicco C, Al Khodor S, Bilinski J, Caselli E, Comelli EM, Egert M, Giaroni C, Karpinski TM, Loniewski I, Mulak A, Reygner J, Samczuk P, Serino M, Sikora M, Terranegra A, Ufnal M, Villeger R, Pichon C, Konturek P, Edeas M. 2022. Microbiota medicine: towards clinical revolution. J Transl Med 20:111. doi:10.1186/s12967-022-03296-9 35255932 PMC8900094

[57] Verdier C, Denis S, Gasc C, Boucinha L, Uriot O, Delmas D, Dore J, Le Camus C, Schwintner C, Blanquet-Diot S. 2021. An oral FMT capsule as efficient as an enema for microbiota reconstruction following disruption by antibiotics, as assessed in an in vitro human gut model. Microorganisms 9:358. doi:10.3390/microorganisms9020358 33670255 PMC7918368

[58] Ramai D, Zakhia K, Ofosu A, Ofori E, Reddy M. 2019. Fecal microbiota transplantation: donor relation, fresh or frozen, delivery methods, cost-effectiveness. Ann Gastroenterol 32:30–38. doi:10.20524/aog.2018.0328 30598589 PMC6302197

[59] Manor O, Dai CL, Kornilov SA, Smith B, Price ND, Lovejoy JC, Gibbons SM, Magis AT. 2020. Health and disease markers correlate with gut microbiome composition across thousands of people. Nat Commun 11:5206. doi:10.1038/s41467-020-18871-1 33060586 PMC7562722

[60] Średnicka P, Roszko MŁ, Popowski D, Kowalczyk M, Wójcicki M, Emanowicz P, Szczepańska M, Kotyrba D, Juszczuk-Kubiak E. 2023. Effect of in vitro cultivation on human gut microbiota composition using 16S rDNA amplicon sequencing and metabolomics approach. Sci Rep 13:3026. doi:10.1038/s41598-023-29637-2 36810418 PMC9945476

[61] Mendelson M, Matsoso MP. 2015. The world health organization global action plan for antimicrobial resistance. S Afr Med J 105:325. doi:10.7196/samj.9644 26242647

[62] Wang X, Xing Y, Ji Y, Xi H, Liu X, Yang L, Lei L, Han W, Gu J. 2022. The combination of phages and faecal microbiota transplantation can effectively treat mouse colitis caused by Salmonella enterica serovar Typhimurium. Front Microbiol 13:944495. doi:10.3389/fmicb.2022.944495 35875536 PMC9301289

[63] Bhutani D, Jaiyeoba C, Kim S, Naylor P, Uberti JP, Ratanatharathorn V, Ayash L, Deol A, Alavi A, Revankar S, Chandrasekar P. 2019. Relationship between Clostridium difficile infection and gastrointestinal graft versus host disease in recipients of allogeneic stem cell transplantation. Bone Marrow Transplant 54:164–167. doi:10.1038/s41409-018-0270-x 30038352 PMC6844071

[64] Antharam VC, Li EC, Ishmael A, Sharma A, Mai V, Rand KH, Wang GP. 2013. Intestinal dysbiosis and depletion of butyrogenic bacteria in Clostridium difficile infection and nosocomial diarrhea. J Clin Microbiol 51:2884–2892. doi:10.1128/JCM.00845-13 23804381 PMC3754663

[65] Hamilton MJ, Weingarden AR, Unno T, Khoruts A, Sadowsky MJ. 2013. High-throughput DNA sequence analysis reveals stable engraftment of gut microbiota following transplantation of previously frozen fecal bacteria. Gut Microbes 4:125–135. doi:10.4161/gmic.23571 23333862 PMC3595072

[66] Seekatz AM, Aas J, Gessert CE, Rubin TA, Saman DM, Bakken JS, Young VB. 2014. Recovery of the gut microbiome following fecal microbiota transplantation. mBio 5:e00893-14. doi:10.1128/mBio.00893-14 24939885 PMC4068257

[67] Theriot CM, Young VB. 2015. Interactions between the gastrointestinal microbiome and Clostridium difficile. Annu Rev Microbiol 69:445–461. doi:10.1146/annurev-micro-091014-104115 26488281 PMC4892173

[68] Ankersen DV, Weimers P, Marker D, Johannesen T, Iversen S, Lilje B, Kristoffersen AB, Saboori S, Paridaens K, Skytt Andersen P, Burisch J, Munkholm P. 2020. eHealth: disease activity measures are related to the faecal gut microbiota in adult patients with ulcerative colitis. Scand J Gastroenterol 55:1291–1300. doi:10.1080/00365521.2020.1829031 33045169

[69] Fachi JL, Felipe J de S, Pral LP, da Silva BK, Corrêa RO, de Andrade MCP, da Fonseca DM, Basso PJ, Câmara NOS, de Sales E Souza ÉL, Dos Santos Martins F, Guima SES, Thomas AM, Setubal JC, Magalhães YT, Forti FL, Candreva T, Rodrigues HG, de Jesus MB, Consonni SR, Farias ADS, Varga-Weisz P, Vinolo MAR. 2019. Butyrate protects mice from Clostridium difficile-induced colitis through an HIF-1-dependent mechanism. Cell Rep 27:750–761. doi:10.1016/j.celrep.2019.03.054 30995474

[70] Sorg JA, Sonenshein AL. 2008. Bile salts and glycine as cogerminants for Clostridium difficile spores. J Bacteriol 190:2505–2512. doi:10.1128/JB.01765-07 18245298 PMC2293200

[71] Gupta S, Allen-Vercoe E, Petrof EO. 2016. Fecal microbiota transplantation: in perspective. Therap Adv Gastroenterol 9:229–239. doi:10.1177/1756283X15607414 PMC4749851 26929784

[72] Fijan S, Šulc D, Steyer A. 2018. Study of the in vitro antagonistic activity of various single-strain and multi-strain probiotics against Escherichia coli. Int J Environ Res Public Health 15:1539. doi:10.3390/ijerph15071539 30036977 PMC6069398

[73] Schmidt TSB, Li SS, Maistrenko OM, Akanni W, Coelho LP, Dolai S, Fullam A, Glazek AM, Hercog R, Herrema H, Jung F, Kandels S, Orakov A, Thielemann R, von Stetten M, Van Rossum T, Benes V, Borody TJ, de Vos WM, Ponsioen CY, Nieuwdorp M, Bork P. 2022. Drivers and determinants of strain dynamics following fecal microbiota transplantation. Nat Med 28:1902–1912. doi:10.1038/s41591-022-01913-0 36109636 PMC9499871

[74] Ianiro G, Punčochář M, Karcher N, Porcari S, Armanini F, Asnicar F, Beghini F, Blanco-Míguez A, Cumbo F, Manghi P, Pinto F, Masucci L, Quaranta G, De Giorgi S, Sciumè GD, Bibbò S, Del Chierico F, Putignani L, Sanguinetti M, Gasbarrini A, Valles-Colomer M, Cammarota G, Segata N. 2022. Variability of strain engraftment and predictability of microbiome composition after fecal microbiota transplantation across different diseases. Nat Med 28:1913–1923. doi:10.1038/s41591-022-01964-3 36109637 PMC9499858

[75] Wilson BC, Vatanen T, Jayasinghe TN, Leong KSW, Derraik JGB, Albert BB, Chiavaroli V, Svirskis DM, Beck KL, Conlon CA, Jiang Y, Schierding W, Holland DJ, Cutfield WS, O’Sullivan JM. 2021. Strain engraftment competition and functional augmentation in a multi-donor fecal microbiota transplantation trial for obesity. Microbiome 9:107. doi:10.1186/s40168-021-01060-7 33985595 PMC8120839

[76] Chehri M, Christensen AH, Halkjær SI, Günther S, Petersen AM, Helms M. 2018. Case series of successful treatment with fecal microbiota transplant (FMT) oral capsules mixed from multiple donors even in patients previously treated with FMT enemas for recurrent Clostridium difficile infection. Medicine (Baltimore) 97:e11706. doi:10.1097/MD.0000000000011706 30075573 PMC6081131

[77] Kedia S, Virmani S, K Vuyyuru S, Kumar P, Kante B, Sahu P, Kaushal K, Farooqui M, Singh M, Verma M, Bajaj A, Markandey M, Sachdeva K, Das P, Makharia GK, Ahuja V. 2022. Faecal microbiota transplantation with anti-inflammatory diet (FMT-AID) followed by anti-inflammatory diet alone is effective in inducing and maintaining remission over 1 year in mild to moderate ulcerative colitis: a randomised controlled trial. Gut 71:2401–2413. doi:10.1136/gutjnl-2022-327811 35973787

[78] Cold F, Browne PD, Günther S, Halkjaer SI, Petersen AM, Al-Gibouri Z, Hansen LH, Christensen AH. 2019. Multidonor FMT capsules improve symptoms and decrease fecal calprotectin in ulcerative colitis patients while treated – an open-label pilot study. Scand J Gastroenterol 54:289–296. doi:10.1080/00365521.2019.1585939 30946615

[79] Guo X-H, Zhu Y-L, Yang L, Li W-J, Du X-F. 2022. The effects of multi-donor fecal microbiota transplantation capsules combined with thalidomide on hormone-dependent ulcerative colitis. Infect Drug Resist 15:7495–7501. doi:10.2147/IDR.S385485 36570710 PMC9784385

[80] Shou D, Luo Q, Tang W, Cao C, Huang H, Chen H, Zhou Y. 2023. Hepatobiliary and pancreatic: multi-donor fecal microbiota transplantation attenuated high-fat diet-induced hepatic steatosis in mice by remodeling the gut microbiota. J Gastroenterol Hepatol 38:2195–2205. doi:10.1111/jgh.16359 37787118

