# Supplementary material for: Reduction of product composition variability using pooled microbiome ecosystem therapy and consequence in two infectious murine models
Source: Appl Environ Microbiol. 2024 Apr 23;90(5):e00016-24. doi: 10.1128/aem.00016-24 (PMC11107171; doi:10.1128/aem.00016-24)
Supplement: Supplemental tables and figures — Table S1; Figures S1 and S2. [file aem.00016-24-s0001.docx]

**Supplemental Material**

Table S1: Summary of the human fecal product batches used in the study.

| **set** | **Product** | ***In vivo*** | ***In vitro*** |
| --- | --- | --- | --- |
| **1** | **Pool A** | **Yes** | **Yes** |
|  | **donor 1** |  |  |
|  | **donor 2** |  |  |
|  | **donor 3** |  |  |
|  | **donor 4** |  |  |
|  | **donor 5** |  |  |
| **2** | **Pool B** | **No** | **No** |
|  | **donor 6** |  |  |
|  | **donor 7** |  |  |
|  | **donor 8** |  |  |
|  | **donor 9** |  |  |
|  | **donor 10** |  |  |
| **3** | **Pool C** | **No** | **Yes** |
|  | **donor 11** |  |  |
|  | **donor 12** |  |  |
|  | **donor 13** |  |  |
|  | **donor 14** |  |  |
|  | **donor 15** |  |  |
|  | **donor 16** |  |  |
|  | **donor 17** |  |  |
| **4** | **Pool D** | **No** | **Yes** |
|  | **donor 18** |  |  |
|  | **donor 19** |  |  |
|  | **donor 20** |  |  |
|  | **donor 21** |  |  |
|  | **donor 22** |  |  |
|  | **donor 23** |  |  |
|  | **donor 24** |  |  |
|  | **donor 25** |  |  |
| **5** | **Pool E** | **No** | **Yes** |
|  | **donor 26** |  |  |
|  | **donor 27** |  |  |
|  | **donor 28** |  |  |
|  | **donor 29** |  |  |
|  | **donor 30** |  |  |
|  | **donor 44** |  |  |
| **6** | **Pool F** | **No** | **Yes** |
|  | **donor 45** |  |  |
|  | **donor 46** |  |  |
|  | **donor 31** |  |  |
|  | **donor 32** |  |  |
|  | **donor 33** |  |  |
|  | **donor 34** |  |  |
|  | **donor 47** |  |  |
|  | **donor 35** |  |  |
|  | **donor 36** |  |  |
| **7** | **Pool G** | **Yes** | **No** |
|  | **donor 37** |  |  |
|  | **donor 48** |  |  |
|  | **donor 38** |  |  |
|  | **donor 39** |  |  |
|  | **donor 40** |  |  |
|  | **donor 41** |  |  |
|  | **donor 42** |  |  |
|  | **donor 43** |  |  |


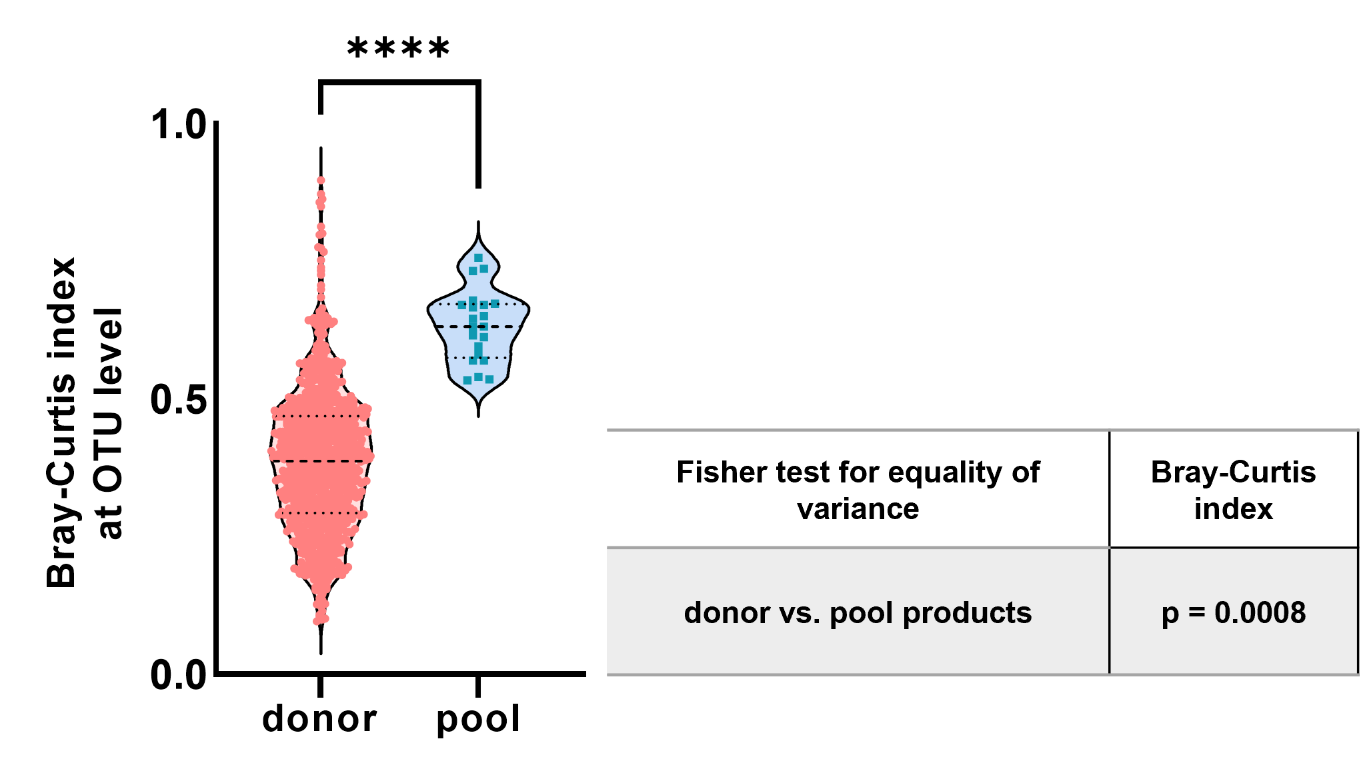


Figure S1. Bray-Curtis similarity index of donor-derived products and pooled products. Unpaired t test was used (p < 0.0001) and Fisher test for equality of variance was performed.


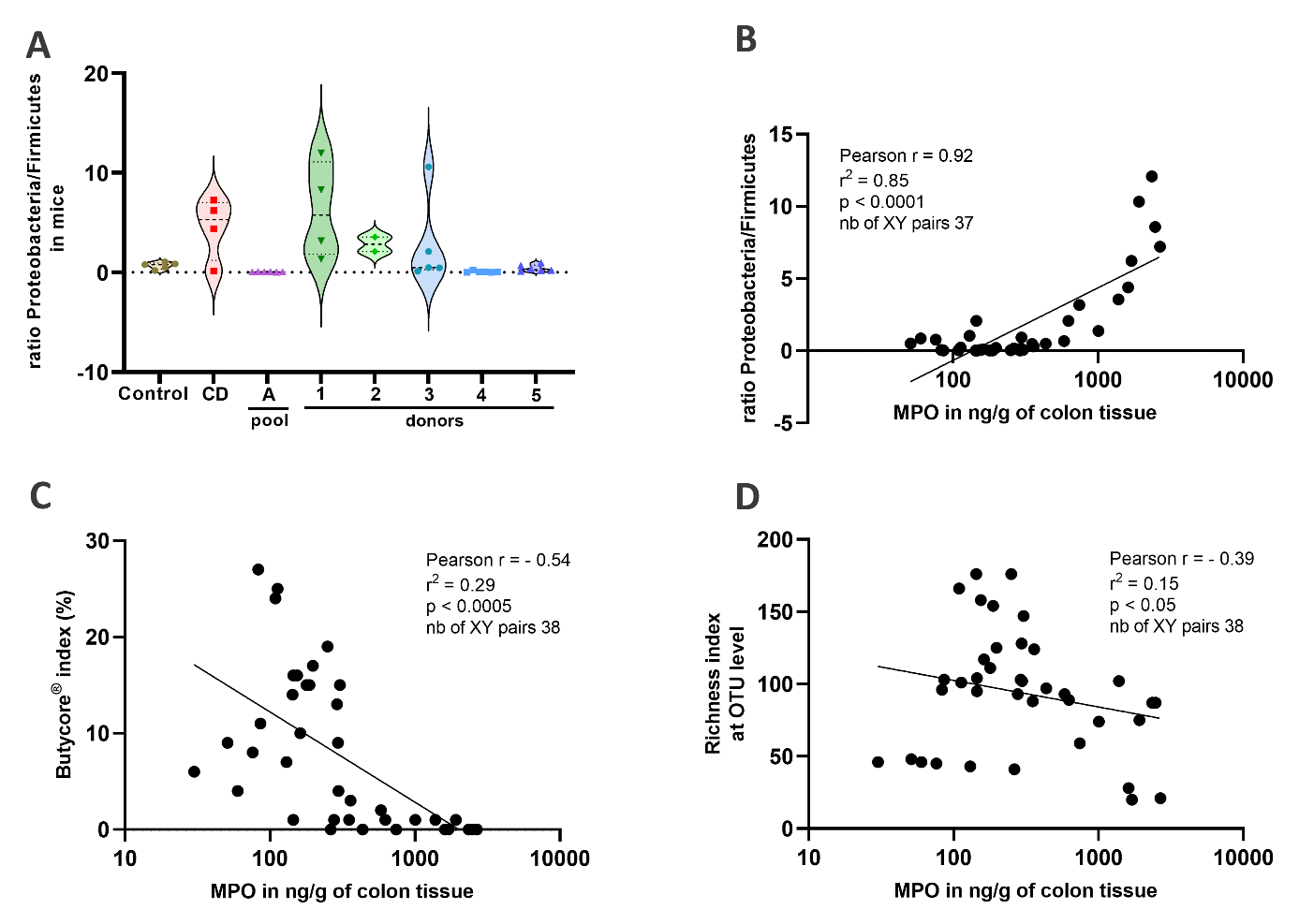


Figure S2. Microbiome 16S rDNA description and correlation analysis.

(A) Proteobacteria/Firmicutes ratio in mice feces. (B) Pearson correlation analysis between MPO level and Proteobacteria/Firmicutes ratio. (C) Pearson correlation analysis between MPO level and Butycore® index. (D) Pearson correlation analysis between MPO level and richness at OTU level.
